# Predicting Protein–protein Association Rates using Coarse-grained Simulation and Machine Learning

**DOI:** 10.1038/srep46622

**Published:** 2017-04-18

**Authors:** Zhong-Ru Xie, Jiawen Chen, Yinghao Wu

**Affiliations:** 1 Department of Systems and Computational Biology, Albert Einstein College of Medicine, Yeshiva University, 1300 Morris Park Avenue, Bronx, NY, 10461, USA

## Abstract

Protein–protein interactions dominate all major biological processes in living cells. We have developed a new Monte Carlo-based simulation algorithm to study the kinetic process of protein association. We tested our method on a previously used large benchmark set of 49 protein complexes. The predicted rate was overestimated in the benchmark test compared to the experimental results for a group of protein complexes. We hypothesized that this resulted from molecular flexibility at the interface regions of the interacting proteins. After applying a machine learning algorithm with input variables that accounted for both the conformational flexibility and the energetic factor of binding, we successfully identified most of the protein complexes with overestimated association rates and improved our final prediction by using a cross-validation test. This method was then applied to a new independent test set and resulted in a similar prediction accuracy to that obtained using the training set. It has been thought that diffusion-limited protein association is dominated by long-range interactions. Our results provide strong evidence that the conformational flexibility also plays an important role in regulating protein association. Our studies provide new insights into the mechanism of protein association and offer a computationally efficient tool for predicting its rate.

Protein interactions constitute an indispensable part of all cellular processes^1^,^2^,^3^,^4^,^5^,^6^, and strong interactions between protein subunits drive the assembly of permanent molecular machines, such as ATP synthase^7^,^8^,^9^, and regulate the formation of transient protein complexes in cell signaling pathways^10^. This thermodynamic property of protein interactions is characterized by dissociation constants (*K*_*d*_) that quantitatively determine the stability of a protein complex after binding^11^. In addition to the *K*_*d*,_ the kinetic aspect of binding (i.e., how fast two proteins associate) is usually as important to the biological functions of proteins in cells^12^,^13^ as the thermodynamics. For instance, the binding kinetics between membrane receptors and their ligands control the speed of signal transduction after cells are exposed to stimulation^14^. Moreover, any cellular activity, such as transcriptional regulation, involves the coordinated effects of several different proteins^15^. The temporal patterns of these dynamic systems are determined by the kinetic information for all pairwise interactions in complicated networks, and the processes of association and dissociation between two proteins are therefore topics of intense study. In principle, the relationship between the rate of association, *k*_*on*_, and the rate of dissociation, *k*_*off*_, is defined by *K*_*d*_ = *k*_*off*_/*k*_*on*_, in which *k*_*on*_ and *k*_*off*_ have units of M^−1^ s^−1^ and s^−1^, respectively, if a first-order reaction is considered in which one ligand only binds to one receptor. The values of *k*_*on*_ and *k*_*off*_ can be experimentally measured using a number of *in vitro* and *in vivo* methods. *In vitro* biophysical techniques, such as analytical ultracentrifugation (AUC)^16^, NMR spectroscopy^17^, isothermal titration calorimetry (ITC)^18^,^19^, surface plasmon resonance (SPR)^20^, and mass spectrometry^21^, allow the quantitative analysis of the stoichiometry or binding parameters of complexes but lose the biological relevance of the binding processes^22^. By contrast, *in vivo* approaches such as cross-linking^23^, Forster resonance energy transfer (FRET)^24^, and fluorescence recovery after photobleaching (FRAP)^25^ can be used to detect the binding of proteins in their physiological environments. However, the kinetic information that they can provide is relatively incomplete due to the multiple levels of cellular complexity. Surprisingly, the observed values for the *k*_*on*_ span an extremely wide range: between 1 M^−1^ s^−1^ and 10^10^ M^−1^ s^−1^ 26,27,28,29,30,31,32. Multiple factors, such as diffusion, the binding energy, and the conformational flexibility, are thought to account for this ten order of magnitude difference in the *k*_*on*_^4^. However, the traditional experimental approaches do not provide mechanistic details of protein association^22^, thus preventing a quantitative understanding of the problem.

A number of different models for the mechanism of protein association have been proposed. The earliest proposed mechanism was the lock-and-key model, in which binding was described as rigid-body docking with surface complementarity. In an alternative approach, known as the induced-fit model^33^, binding triggers a shift in the conformation of a protein from an unbound state to a bound state^34^. This was followed by the conformational selection model^35^,^36^, in which a protein remains in a pre-existing equilibrium of unbound conformations, and binding shifts the equilibrium toward its bound state. Computational approaches have unique advantages over experimental studies for testing the validity of different mechanisms and allow the testing of conditions that may be difficult or impossible to attain in the laboratory. Consequently, a variety of computational methods have been developed to calculate the rate constant for protein association. For instance, machine learning techniques have been used to predict association rate constants based on the chemical or structural properties of proteins^37^,^38^. Physics-based methods, such as Brownian dynamic (BD) simulation, are widely used to reproduce the association of two proteins^39^,^40^,^41^,^42^,^43^,^44^,^45^,^46^,^47^,^48^,^49^,^50^,^51^,^52^,^53^,^54^,^55^,^56^,^57^,^58^,^59^,^60^. These all atom-based methods are computationally expensive, as they have to take into account the large amount of freedom in both interacting proteins. Moreover, the role of molecular flexibility implied in the induced fit and conformational selection models is difficult to consider. A more recent method based on BD simulation was proven to successfully predict protein association rate constants using a “transient-complex” theory^61^,^62^,^63^,^64^; this method highlights the importance of electrostatic interactions in protein association and calculates rate constants by decomposing them into energetic and diffusion contributions. However, all the current computational predictions are verified by performing *in vitro* experiments and thus cannot definitively represent the *in vivo* binding of proteins.

The usage of coarse-grained (CG) models is an alternative strategy that enables higher computational efficiency by reducing the size of the simulation system. CG models have been developed to study protein–peptide and protein–protein binding and complex assembly^65^,^66^,^67^. In this article, we develop a new CG model to simulate the process of protein association using the kinetic Monte Carlo (KMC) algorithm. Each residue in this model is represented by its Cα atom and the representative center of a side-chain. A simple physics-based force field is used to guide the diffusion of two interacting proteins. For a given size of simulation box and duration of simulation, the association rate constant can be derived by counting the frequency of dimer formation between the two proteins among a large number of simulation trajectories. We tested our method on the wild-type barnase/barstar complex and various mutants^26^,^27^ and on a large benchmark set of 49 protein complexes, the *k*_*on*_ values of which range from 10^4^ to 10^9^ M^−1^ s^−1^. Positive correlations were observed between the experimental measurements and our calculated values, indicating the potential of the method for predicting the rate of protein association. However, the *k*_*on*_ values for some of the protein complexes were overestimated in the benchmark test. Based on the conformational selection model, we hypothesized that this overestimation resulted partially from the molecular flexibility at the interface regions of the interacting proteins. After inputting variables, including the percentage of flexible loop residues from each protein at the binding surfaces to take into account the impact of the molecular flexibility using a machine learning algorithm, we successfully distinguished the most overestimated association rates from the non-overestimated ones and were thus able to correct the overestimated rate constants and improve the final prediction in a cross-validation test set. This method, which, to the best of our knowledge, is the first to combine physics-based simulation and a machine-learning algorithm, was then applied to a newly constructed independent 10 complex test set, and a strong correlation was obtained between our predicted *k*_*on*_ values and the experimentally measured values. It was thought that the protein association in a diffusion-limited system is dominated by long-range interactions at the binding interfaces^4^. However, our results provide strong evidence that the conformational flexibility of protein structures plays a broader role in regulating the protein association than previously anticipated. In summary, our study provides new insights into the mechanism of protein association and provides a computationally efficient tool for predicting its rate.

## Results

### Testing the robustness of the KMC simulation for calculating the protein association rate

We used the association of the proteins barnase and barstar as a test system to evaluate the robustness of our KMC simulation. The barnase/barstar complex (PDB id 1BRS) was separated into two monomers and randomly placed in a 10 × 10 × 10 nm cubic simulation box. The parameter ξ (Coulomb Debye length) in the simulation was 9.5 Å, which corresponded to an ionic strength of 103 mM. The relation between ξ and the ionic strength will be discussed in the next section. Starting from a random orientation of two monomers, 10^4^ simulation trajectories with a maximal duration of 1000 ns were generated, and encounter complexes were observed in 658 of these, giving a success rate, ρ, of 0.0658. Knowing the volume of the simulation box, the maximal duration of each trajectory, and the success rate, equation (8) was then used to calculate the *k*_*on*_, which was 4.22 × 10^7^ M^−1^ s^−1^. This result, which is close to the experimental measurement (1.2 × 10^8^ M^−1^ s^−1^) at the ionic strength of 103 mM, indicates a fast association between these two proteins.

**Figure 1:**
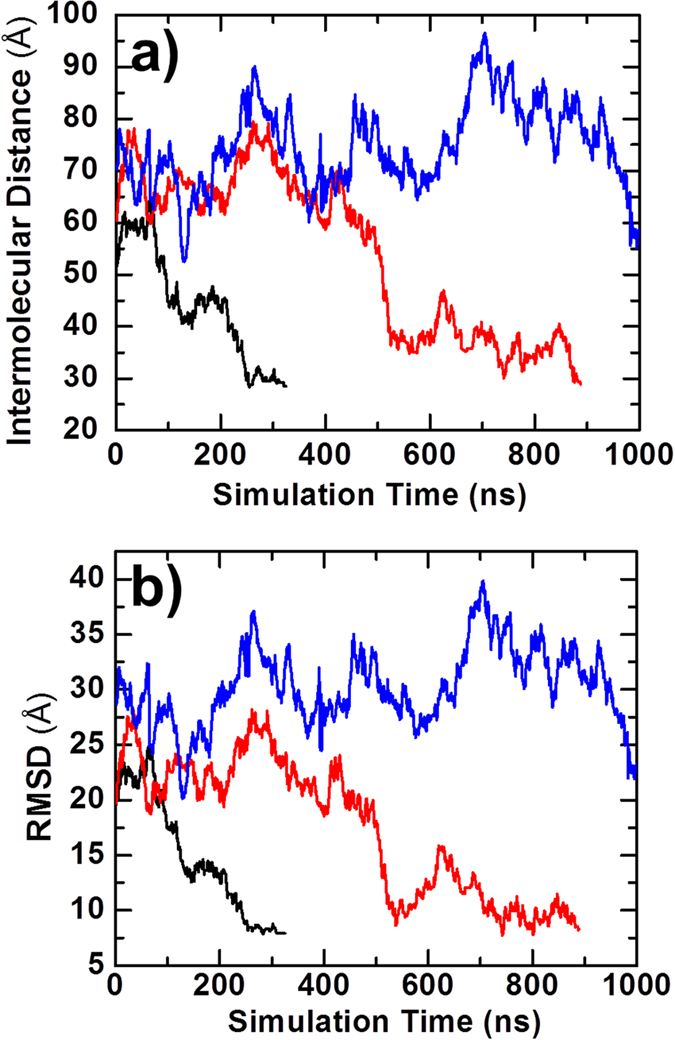
The association of the proteins barnase and barstar was first used as a test system. The complex was separated into two monomers and randomly placed in a 10 × 10 × 10 nm cubic simulation box. In total, 10^4^ simulation trajectories with a maximal duration of 1000 ns were generated, and each trajectory was terminated upon the formation of an encounter complex. Three representative trajectories are plotted to illustrate how the distance between the centers of mass for the two monomers (**a**) and the RMSD from the native complex (**b**) changed with the simulation time.

Three representative trajectories are selected to illustrate the physical process of association in simulations. The changes of distance between two monomers’ centers of mass are plotted in Fig. 1a with the simulation time, while the changes in the RMSD from the native complex are plotted in Fig. 1b. The inter-molecular distance and RMSD are large at the beginning of the simulations, given the initial random conformations in all three trajectories. The figure shows that the proteins associated into complexes faster in some trajectories than others. For instance, the complex in the black curve was formed at 300 ns, whereas the complex in the red curve was formed at 900 ns. In these cases, the proteins diffused around in the simulation box and spatially approached each other until they found their actual binding sites. However, in some cases, the proteins cannot associate into complexes by the end of the maximal time duration (blue curve). Therefore, large diversity exists among each individual trajectory. The final meaningful calculation of *k*_*on*_ cannot be derived without the statistical analysis of all 10^4^ trajectories.

**Figure 2:**
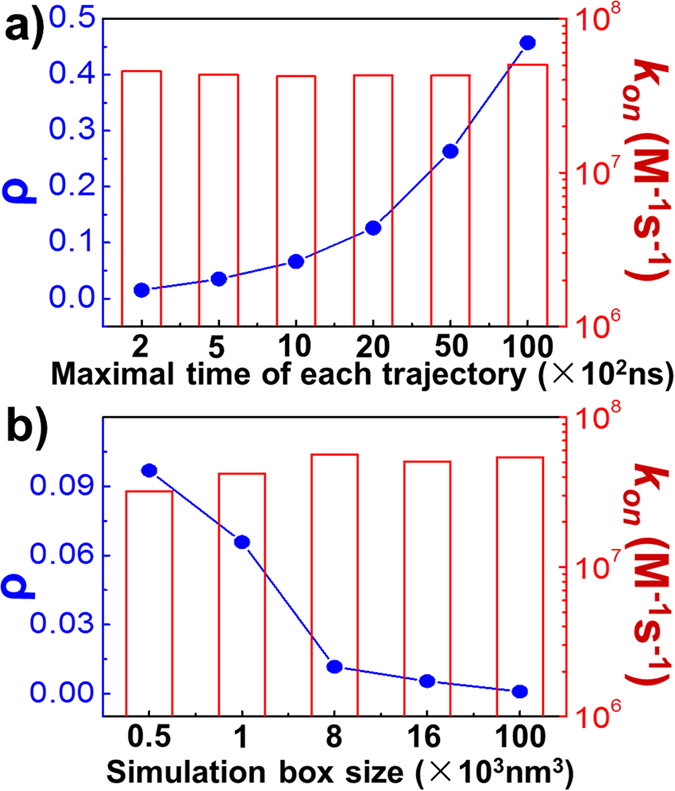
(**a**) Effect of changing the maximal duration of each simulation trajectory on the success rate (ρ). Simulations were performed in a 10 × 10 × 10 nm cubic box. (**b**) Effect of changing the size of the simulation box on the success rate. The maximal duration of the simulation time for each trajectory was 1000 ns.

We then changed the maximal duration of each simulation trajectory. As shown in Fig. 2a, an increase in the maximal duration led to a higher success rate (blue dots and line), indicating that, given sufficient time, two proteins have a higher probability of association. By contrast, the calculated *k*_*on*_ values, shown by the red bars in Fig. 2a, were very consistent at different simulation durations. As shown in equation (8), the calculated *k*_*on*_ was normalized by the maximal duration of the simulation and is thus temporally insensitive. We also tried simulation boxes of different sizes. As shown in Fig. 2b, larger simulation boxes resulted in lower success rates, i.e., the diffusion of proteins in a larger volume causes association to occur more slowly. The success rates for different volumes were then used to calculate the corresponding *k*_*on*_ values. Overall, the calculated *k*_*on*_ values were relatively stable in large volumes, which suggested that the size of the simulation box had little effect on our prediction results under relatively low concentrations. However, the *k*_*on*_ for a small-volume box was relatively low because the increasing nonspecific interactions at a high concentration hinder the proper association between two proteins. This effect is not considered in traditional simulation methods, in which the concentrations of the interacting proteins are essentially ignored. Our results suggest that in a crowded cellular environment, the protein association is concentration-dependent. This is consistent with previous studies^68^. In summary, these tests demonstrated that the KMC simulation results were unaffected by the choice of simulation parameters and that this is a robust method for calculating the *k*_*on*_ of protein association.

### Estimating the solvation effect on protein–protein associations

**Figure 3:**
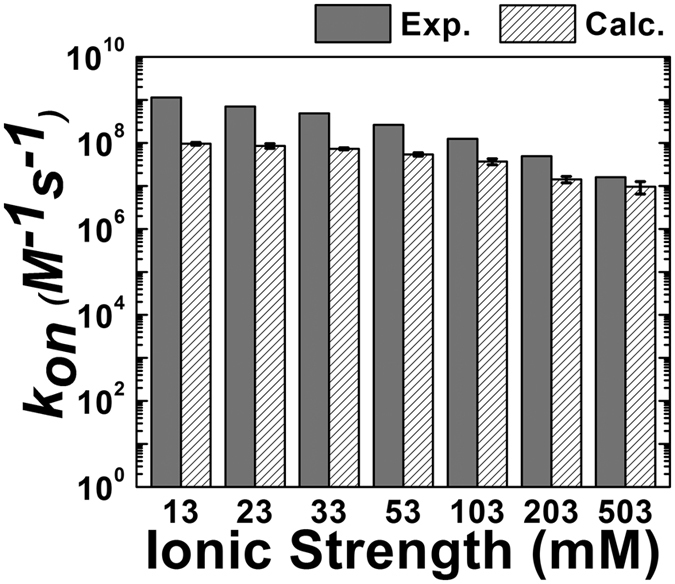
Testing of the effect of the ionic strength on the association of the barnase/barstar complex by changing the Coulomb Debye length in the simulations. The derived *k*_*on*_ values (striped bars with standard deviations) are plotted against different values of ionic strength. Experimental measurements under different values of ionic strength are shown as gray bars. To calculate the standard deviations, 10^4^ KMC simulation trajectories were generated for each value of the specific ionic strength. We randomly divided these trajectories into 10 groups, each containing 10^3^ trajectories. We estimated *k*_*on*_ from the 10^3^ trajectories of each group and derived 10 individual *k*_*on*_ values. The standard deviation was calculated from the group of *k*_*on*_ values.

The concentration of ions around two interacting proteins is an important factor controlling the rate of their association, and the experimentally measured *k*_*on*_ values for protein binding at different ionic strengths show a negative correlation^54^. The salt effect in our CG model is manifested by the Coulomb Debye length, ξ, which describes the decay of the long-range electrostatic interactions between proteins in the solvent. Theoretically, the Coulomb Debye length is related to the ionic strength using the equation^69^


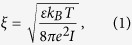


where *k*_*B*_ is the Boltzmann constant, *e* the elementary charge, *I* the ionic strength, *T* the absolute temperature, and ε the solvent medium dielectric constant. In our study, equation (1) was further simplified to 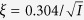 f calculate the Coulomb Debye length at a given ionic strength^70^, and the calculated ξ was then used in the subsequent KMC simulations. We simulated the association of the barnase/barstar complex at ionic strengths of 13, 23, 33, 53, 103, 203, and 503 mM based on the data used in a previous study by Alsallaq and Zhou^61^. The calculated values of ξ at these ionic strengths are listed in Table S1. In accordance with equation (1), the data showed a negative correlation between ξ and the ionic strength. The derived *k*_*on*_ values plotted against the ionic strength are shown in Fig. 3. Although the plot shows that our calculated *k*_*on*_ values were underestimated compared to the experimental measurements, there was qualitative agreement between these two sets of data within the ionic strength range of 50 to 500 mM. The figure shows a fast association at low ionic strength and a slow association at high ionic strength, consistent with previous results^61^. Lower values of ξ shield the long-range electrostatic interactions and therefore slow down the association of proteins at higher ionic strengths^54^. These tests showed that our method can reproduce the effect of the ionic strength on associations of the barnase/barstar complex.

### Evaluating the effects of point mutations on protein association rates

**Figure 4:**
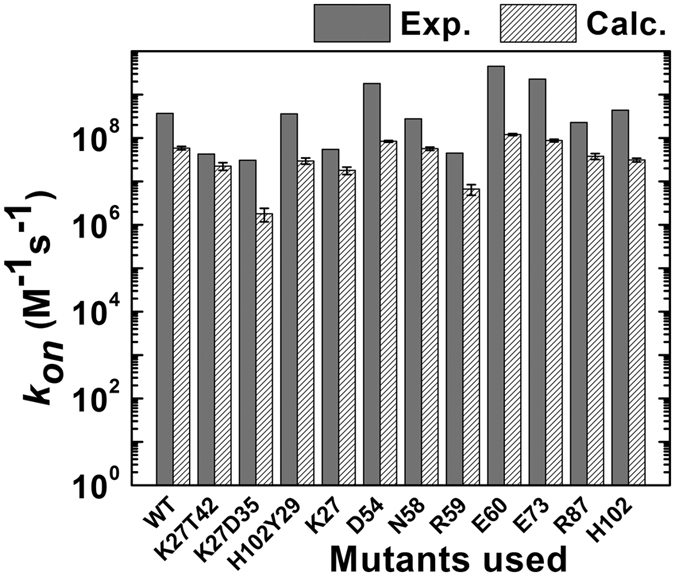
Testing the effect of mutations on the protein association rate (*k*_*on*_). The test set consisted of the wild type of the barnase/barstar protein complex and 11 mutants, in which the indicated residue in barnase (single mutants or the first indicated residue in the double mutants) or barstar (second indicated residue in the double mutants) was mutated to alanine; the mutants are shown below the figure. The experimental measurements are shown as gray bars, and the calculated values as striped bars (with standard deviations).

To systematically validate the sensitivity of our simulation algorithm and scoring function, we tested the effect of protein mutations on the calculated association rates. Mutations of specific residues at binding interfaces change the interactions between proteins, affecting their association rates. Our test set consisted of the wild type barnase/barstar protein complex plus 11 complexes of barnase mutants and wild-type barstar or complexes of mutants of both proteins in which the target amino acid(s) were mutated into alanine; the experimental *k*_*on*_ values for these complexes have been reported in a previous study^27^. The side chains of the corresponding residues were computationally replaced for each mutant before the KMC simulation of its association (see Model and Methods). Figure 4 shows a comparison of the predicted *k*_*on*_ values for these mutants (striped bars) and the experimental values (gray bars) at an ion concentration of 50 mM^27^; the sequence number of the mutated residue is shown on the x axis, with the 8 single mutations being in barnase, while in the case of the double mutants, the first mutated reside is in barnase and the second in barstar. As shown in the figure, of the eleven mutants, D54, E60, and E73 had the highest calculated *k*_*on*_ values (higher than that for the wild-type complex), while K27D35 and R59 had the lowest.

Figure 4 shows that, overall, the calculated *k*_*on*_ values were underestimated compared to the experimental values. However, our computational model was able to reproduce the relative order of the rate constants for the mutant complexes compared to that for the wild-type complex. For instance, our simulations showed that the mutation of D54, E60, or E73 to alanine accelerated the association, while the mutation of K27D35 or R59 to alanine decreased the association. This result therefore suggests that for the barnase/barstar complex, our model can capture the effects of single- and double-point mutations on the association rates.

### Validation of the accuracy of the KMC simulation using a large-scale benchmark set

**Figure 5:**
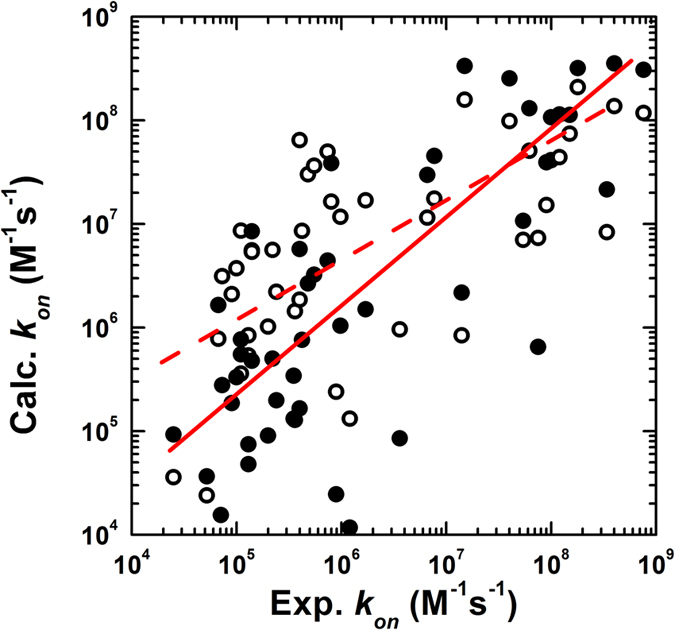
Testing of the KMC simulations on a large benchmark set of 47 protein complexes by comparing the calculated and observed log_10_ *k*_*on*_ values (white circles), giving a Pearson’s correlation coefficient of 0.66. However, the calculated association rates for a large percentage of the protein complexes were significantly overestimated, so a machine learning algorithm was used to recognize these overestimated cases and correct the corresponding *k*_*on*_ values by an adjustment factor. After applying a leave-one-out cross-validation test, the Pearson’s correlation coefficient between the log_10_ values for the adjusted *k*_*on*_ values and their experimental values (black circles) was 0.79. The dashed red line is from linear regression fit between simulated and observed log_10_ *k*_*on*_ values, with a slope of 0.52 and intercept of 3.39. The solid red line is from linear regression fit between adjusted and observed log_10_ *k*_*on*_ values, with a slope of 0.8 and intercept of 1.32.

To test the generality of our KMC simulation method, we used a large-scale benchmark set of 49 protein complexes for which experimental measurements of the *k*_*on*_ and ionic strength have been reported previously^64^. Detailed information about the benchmark set can be found in the Methods and Supporting Information Table S2A. For each complex in the benchmark set, 10^4^ simulation trajectories were carried out based on the reported ionic strengths and native structures listed in Table S2A. Each trajectory has a maximal duration of 1000 ns and was initiated starting from a random orientation in which two monomers were placed in a 10 × 10 × 10 nm cubic simulation box. The *k*_*on*_ values were calculated based on the simulations for all the 49 complexes except two (3BP8 and 1VFB), for which the simulations did not generate any output. This could be because a multistep association mechanism was involved. Figure 5 shows a log base 10 plot of our calculated data and the experimental data for the remaining 47 complexes, shown as white dots, with a Pearson’s correlation coefficient of 0.66. This positive correlation between the calculated results and experimental data indicates that the combination of a simple physics-based scoring function and a CG simulation algorithm can distinguish between the fast and slow kinetics of a wide range of protein–protein associations.

Most previous studies of all-atom BD simulations were tested only on a few individual cases. The reaction criteria in these all-atom simulations are determined by a structural parameter, *Q*, which is defined as the number of intermolecular native contacts formed during the simulations divided by the total number of contacts that would be found in the final complex. These criteria were normally varied in different studies, or even in the same study, to achieve the best agreement with the experimental results^12^,^45^. In contrast to previous BD simulation studies, we used the same criteria (at least 3 native connections restored and an rmsd < 10 Å) for all the protein complexes. Our CG model thus offers a general predictive method for calculating protein association rates based on physical principles. The same benchmark set was tested by *TransComp*, which is based on the “transient-complex” theory^61^,^62^,^63^,^64^ and all-atom BD simulations. The association rate constant in *TransComp* is calculated as 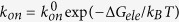, where 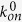 is the basal rate constant for reaching the transient complex by random diffusion and Δ*G*_*ele*_ is the electrostatic interaction free energy of the transient complex. Comparing the *k*_*on*_ values predicted by *TransComp* with those calculated from our KMC simulations, we observed that, in some cases, the *k*_*on*_ values calculated by our model are closer to the experimental values than those predicted by *TransComp*. For example, for the complex CheY/CheA (PDB id 1FFW, experimental kon equals 6.2 × 10^7^ M^−1^ s^−1^), our calculated value (5.8 × 10^7^ M^−1^ s^−1^) is more accurate than that of *TransComp* (9.0 × 10^6^ M^−1^ s^−1^). In some cases, on the other hand, *k*_*on*_ values calculated by our model are less accurate than those of *TransComp*. For instance, the experimental *k*_*on*_ of the complex Mlc transcription regulator/EIICB (PDB id 3BP8) is 1.0 × 10^6^ M^−1^ s^−1^, which is closer to that of *TransComp* (6.3 × 10^6^ M^−1^ s^−1^) than that of our simulation (3.0 × 10^4^ M^−1^ s^−1^). The underestimation by KMC for this specific system is possibly due to the small binding interface in this complex. Starting from a random initial configuration, the native-like structure of this complex is relatively difficult to be sampled by the KMC simulation. It is also worth mentioning that the initial *TransComp* runs were not able to generate results for a few cases that contain extended interfaces in the native complexes, such as streptokinase/plasmin (PDB id 1BML) and thrombin/hirudin (PDB id 4HTC). To compare with the experiments in these cases, partial structures of the protein complexes were chosen as inputs. Our KMC simulations were able to produce reasonably accurate *k*_*on*_ values for these cases. Moreover, we carried out a blinder benchmark test in which the full-length proteins of all 49 complexes were used in our CG simulations. As shown in the next section, the correlation with experimental measurements could be increased by integrating a machine learning-based module to identify potentially overestimated calculated results. Future upgrades of our Kinetic Monte-Carlo simulation are also proposed in the Discussion.

### Integration of a machine learning-based correction module to improve the results for the benchmark test

One major factor affecting the correlation with the experimental measurements was that the *k*_*on*_ values calculated from the KMC simulations were overestimated for a large percentage of protein complexes in the benchmark set. Interestingly, we are not the first to observe this phenomenon. Gabdoulline and Wade^44^ reported BD simulation results for five protein complexes and found that the *k*_*on*_ values for three protein complexes were accurately reproduced, while those for the other two protein complexes were significantly overestimated by approximately 30-fold. The authors proposed that this may have been due to protein flexibility, suggesting that the flexibility of the secondary structure of the proteins at a binding interface may be related to the *k*_*on*_ overestimation in many simulation models. Because the intramolecular degrees of freedom were fixed in our KMC simulations, the structural flexibility is also one of the factors that we did not take into account in the calculation of *k*_*on*_. This implies that the lack of intramolecular flexibility in the simulation might be one of reasons that led to the overestimation of the calculated *k*_*on*_ values. Other factors, such as the electrostatic interactions between residues that are not at binding interfaces, could also cause non-specific interactions that interfere with the association rates. If we were able to identify the structural flexibility and other factors that are responsible for the overestimation and use them to identify protein complexes with overestimated *k*_*on*_ values, we would be able to not only improve predictions purely based on KMC simulations but also better understand the molecular mechanisms of protein association.

Based on the results from our KMC simulations and similar observations in the study by Gabdoulline and Wade, we hypothesized that even when the protein association is dominated by diffusion, it is regulated by a combination of structural factors (conformational flexibility) and energetic factors (mainly electrostatic interactions). To validate the hypothesis, we applied a proof-of-concept analysis by integrating the elastic network model (ENM)^71^,^72^ into the KMC simulation (Fig. S2). ENM was used to change the conformations of the two interacting proteins during their association. The detailed procedure and results can be found in the Supporting Information. In brief, three protein complexes were primarily tested (Fig. S3). Using the KMC that contains conformational changes, we found that our newly calculated *k*_*on*_ have values that are closer to the experimental values. Especially in the case of 1GXD, for which the *k*_*on*_ was overestimated, we show that the conformational fluctuation due to the high structural flexibility can impede association. Unfortunately, ENM has difficulty in modeling large conformational changes due to its limitation of using the harmonic approximation of the force field. Its application to large-scale benchmark tests is under development. No other physics-based method is currently sophisticated enough to fully model the conformational flexibility in simulating protein–protein interactions. Previous methods only considered the effects of conformational changes indirectly by judicially selecting fragments of proteins in a complex as the input structures of simulations.

Therefore, we decided to incorporate the structural flexibility by a different strategy. We added a machine learning-based module to identify and adjust overestimated *k*_*on*_ values. As described in the Methods, we introduced three indicators as inputs for the model. Two of these, the percentages of interface residues on the flexible loops of each of the two interacting proteins, account for the conformational flexibility, whereas the third, the ratio of the electrostatic potential at the binding interface to that of the whole protein pair, accounts for the energetic factor of association, particularly the non-specific interactions. A cross-validation test was then performed on the 47-protein complex benchmark set in which the leave-one-out strategy was applied to avoid potential over-fitting.

The KMC simulations for the 47 complexes in the benchmark set resulted in 23 overestimations and 24 non-overestimations (no outputs for two complexes). Using the leave-one-out training and testing process, we found that the *k*_*on*_ values for 39 of the 47 complexes were predicted correctly as either overestimated or non-estimated, giving an accuracy of 83%. Moreover, of the 23 overestimated cases, 19 were successfully identified, giving a sensitivity of 82.6%. The detailed classification results are shown in Table S3A. After machine learning, all the simulated *k*_*on*_ values were adjusted by a corresponding correction factor based on the classification results; the detailed procedure is described in the Model and Methods. The black circles in Fig. 5 show the correlation between the logarithmic values for the adjusted *k*_*on*_ values and the experimental values for all 47 complexes. In this plot, the Pearson’s correlation coefficient was increased to 0.79 from the original value of 0.66. This improvement resulted from the implementation of the machine learning-based module and highlights the importance of molecular flexibility. We have further performed the linear regression to the dataset. Specifically, the dashed red line in Fig. 5 is from linear regression fit between simulated and observed log_10_*k*_*on*_ values, while the solid red line is from linear regression fit between adjusted and observed log_10_*k*_*on*_ values. Considering the slope of 1 and intercept of 0 in a perfect correlation, the increase of slope and decrease of intercept indicate the prediction results have been improved after the application of machine learning. Thus, the new method combining physics-based simulation with machine learning not only enhanced the predictive potential of our model but also emphasized the functional role of conformational fluctuations, which has been underestimated in the diffusion-limited protein association class.

### Application of the prediction method to a new independent test set

**Figure 6:**
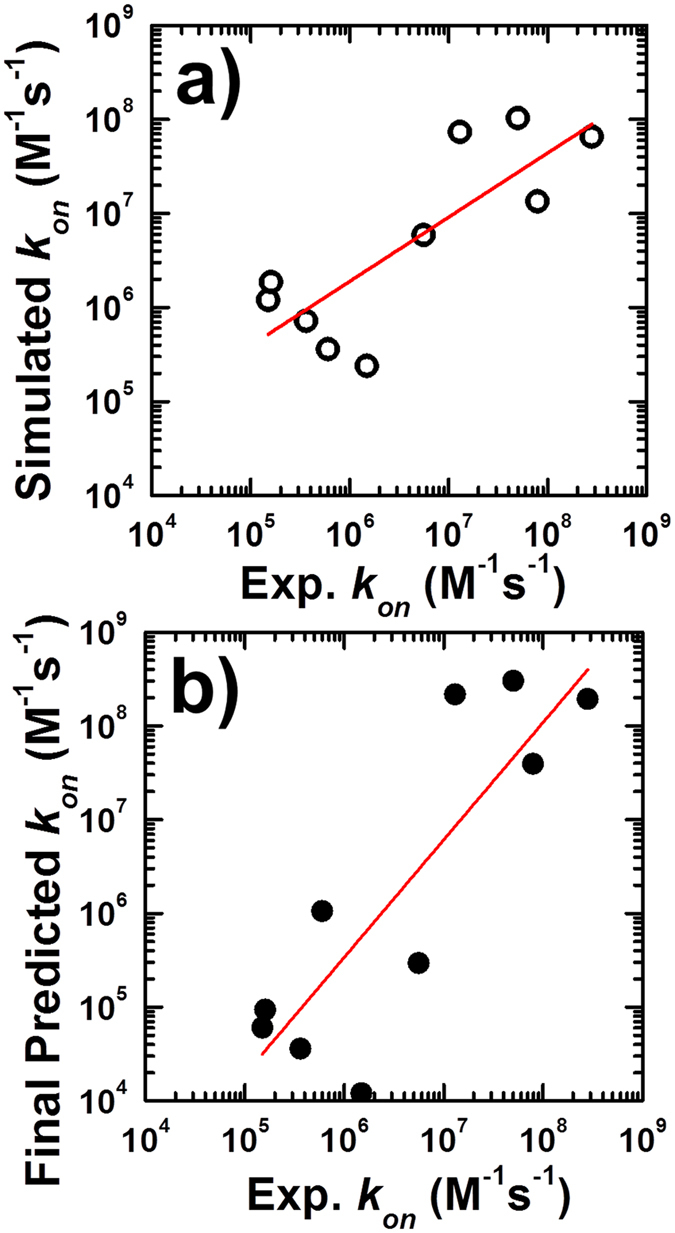
Application of the computational framework to an independent test set. (**a**) The calculated logarithmic values of the *k*_*on*_ from KMC simulations show a high correlation with the experimental data, and the Pearson’s correlation coefficient is 0.8. The red line is from linear regression fit between simulated and observed log_10_*k*_*on*_ values, with a slope of 0.68 and intercept of 2.18. (**b**) The machine learning process was implemented to identify potential overestimation in simulations and adjust the calculated *k*_*on*_ values, giving a Pearson’s correlation coefficient of 0.85. The red line is from linear regression fit between adjusted and observed log_10_*k*_*on*_ values, with a slope of 1.25 and intercept of −1.98.

To further test the stability of our KMC simulations and rule out the possibility of model over-fitting during machine learning, an independent test set of 10 complexes was collected; the detailed information for this set can be found in the Methods and Table S2B. Multiple trajectories were carried out based on the reported ionic strength and corresponding native structure for all 10 protein complexes, starting from the random initial orientations, and then the values of *k*_*on*_ calculated from the simulations were compared to the experimental data, as shown in Fig. 6a. The Pearson’s correlation coefficient between the logarithmic values for the predicted and observed *k*_*on*_ values was 0.8, and this strong correlation indicates the robustness of our KMC method in simulating the rates of protein association. The machine learning process was then applied to the same dataset to identify the potential overestimation in simulations. All 47 of the protein complexes in the previous benchmark set were used as training sets, and each of the 10 protein complexes in the new dataset was individually tested using the trained model. The three indicators for the corresponding protein complex were then input to predict the potential overestimation and the *k*_*on*_ adjusted by the corresponding correction factor. Among the 10 complexes, there are 3 overestimated and 7 non-overestimated k_*on*_s. After our training and testing process, we found that the values for 6 *k*_*on*_s from the 10 complexes were predicted correctly as either overestimated or non-estimated, giving an accuracy of 60%. Moreover, of the 3 overestimated cases, 2 were successfully identified, giving a sensitivity of 66.6%. After the adjustment from machine learning, the final predicted results are plotted in Fig. 6b, which shows that the Pearson’s correlation coefficient between the logarithmic values for the newly predicted and observed *k*_*on*_ values was 0.85. We have also performed the linear regression to this dataset, both before and after the application of machine learning. Specifically, the red line in Fig. 6a shows that the linear regression fit between simulated and observed log_10_*k*_*on*_ values gave a slope of 0.68 and intercept of 2.18. The red line in Fig. 6b shows that the linear regression fit between adjusted and observed log_10_*k*_*on*_ values led to a slope of 1.25 and intercept of −1.98. This suggests that the correlation between predicted and experimental results after machine learning is not significantly improved, but rather over-rectified. The over-rectification is caused by the reason that one data point (3hfm) was misidentified, which significantly shifted the regression result. Moreover, in the dataset with relatively small size, individual cases can cause larger effect on the overall statistical result. Therefore, our study suggest that, although the correlation coefficient after machine learning (0.85) is better than before (0.8), the regression results indicate that a higher correlation coefficient does not necessarily lead to the improvement of prediction results.

Finally, the overall data that combine the 10 complexes with the 47 complexes give a Pearson’s correlation coefficient of 0.78 between the logarithmic values of our predicted and observed *k*_*on*_ values. When the same dataset was tested by the *TransComp* server, the Pearson’s correlation coefficient with the observed *k*_*on*_ values is 0.75. Taken together, our results demonstrate the stability of our computational method for predicting protein association rates and that there is no over-fitting in the training of the machine learning process.

## Discussion

Each cell contains millions of different proteins, the interactions of which maintain the routine functions of the cell^73^,^74^,^75^. In this crowded environment, each protein might bind to more than one target, and different proteins might compete for one binding site. In such cases, the association of a protein with its binding partner is often under kinetic, as well as thermodynamic, control^12^,^13^. Research on the binding kinetics between proteins is thus of paramount importance for understanding their cellular functions^76^. Of the various methods, computational modeling approaches are being intensively studied because they cannot only predict the rate constants of binding but also identify the physical principles governing the association mechanisms. These approaches have been developed based on different disciplines, including machine learning, BD simulations, and transient complex theory, which all depend on an atomic level description of proteins, which is computationally expensive to obtain. In this article, we present a CG method for simulating the process of protein association and calculating the association rate constant. The diffusion of proteins in the simulation is based on a KMC algorithm and is guided by a physical force field to control the kinetics of their association. Applying the KMC simulations, we obtained values for the *k*_*on*_ that were consistent with the experimentally derived values under different simulation conditions, indicating the robustness of our method. Furthermore, after constructing a computational framework that integrated the KMC simulations into a machine learning algorithm, we obtained strong positive correlations between the experimental and predicted *k*_*on*_ values for both a previously used benchmark set of 49 complexes and a newly constructed test set of 10 complexes, indicating the potential of our method as a powerful tool for predicting the *in vitro* protein association rates.

Our computational prediction is based on a physics-based scoring function and Monte Carlo movements to accurately simulate the protein diffusion and conformational changes. This CG model therefore attempts to mimic the biological process of protein association *ab initio*. It has been proposed that the wide spectrum of protein association rate constants can be divided into two groups^5^, those higher than 10^4^ M^−1^ s^−1^, in which the association is limited by protein diffusion, and those lower than this value, in which the association is limited by conformational changes during binding. The significance of the electrostatic complementarity between two binding partners in allowing a fast association in a diffusion-limited system has been previously emphasized. In this diffusion-limited case, the proteins are normally modeled as rigid bodies in the simulation to calculate the association rates. However, in our model, when the conformational flexibility was not considered, we found that a group of protein complexes in the diffusion-limited class had computationally overestimated rate constants. This result is consistent with those of a previous study^44^, in which the predicted rate constants for a small group of wild-type and mutated protein complexes were divided into two classes, in one of which the rate constants were accurately reproduced, but in the other, they were overestimated by a factor of 10 to 30. The conformational selection model of protein–protein binding led us to hypothesize that this overestimation was at least partially caused by the molecular flexibility of different proteins during association, even when the rate constants are for an association in the diffusion-limited class. Using indicators that take into account the secondary structural composition and electrostatic interactions to capture both the conformational and energetic factors of binding, we were able to identify most of the protein complexes with overestimated rate constants and improve the overall prediction results. These data strongly suggest that even the protein association in the diffusion-limited class is co-regulated by multiple factors, and our study therefore adds a new dimension to our understanding of protein association mechanisms.

It has been well accepted that machine learning algorithms are able to provide a mechanistic understanding to biological systems in addition to improving prediction results by adjusting multiple parameters. In terms of protein–protein binding, for example, a feature selection and regression algorithm was applied in a recent study to mine a large set of molecular descriptors about binding interfaces between proteins^38^. This machine-learning-based method used empirical data to construct simple models for the association and dissociation rate constants and then obtained insights from these models. This provided supporting evidence for the conformational selection model in which proteins adopt many shapes, and only those that are in the correct configuration are selected by their binding partner. Similarly, in our study, the machine learning is targeted to explore what was missing in the KMC simulations. The application of machine learning is based on a predefined hypothesis. The input of the machine learning only added one factor that was missing in the original model: structural flexibility. The purpose of the output was to rectify the corresponding error resulting from this model, the systematic overestimation. Through this process, we were able to capture the functional insights of structural flexibility in regulating the protein association.

In our machine learning process, a standard benchmark set containing 49 protein complexes was used for machine learning. We believe that the sample size is large enough for learning in this model. This is due to the following reasons. Firstly, there are only three inputs in our machine learning: the percentage of interface residues on loops of each of the two interacting proteins (factor of flexibility) and the ratio of the electrostatic potential at the binding interface to that of the whole protein pair (factor of energetics). Moreover, the factors of flexibility and energetics are complementary with each other. There is no degeneracy in the space of inputs. At the meanwhile, there are not many other adjustable parameters in the algorithm of the “complex decision tree”. The only parameter is the criterion of overestimation, which has been used based on a previous study^77^. In other words, the size of parameter space in the machine learning is much less than the size of sample size. Furthermore, during machine learning, we tried our best to guarantee that the improvement was not due to the reason of over-fitting through parameter adjustment. As described in Model and Methods, a cross validation test was performed on the 49 protein complex benchmark set in which the leave-one-out strategy was applied to avoid potential over-fitting. Finally, in order to further rule out the possibility of model over-fitting during machine learning, an independent test set was constructed by collecting the most updated experimental data that are not in the standard benchmark set.

Despite the above-mentioned merits, our method has a number of limitations and can be further improved. First, the energy function in our model might be oversimplified, as it only takes into account the most dominant elements in protein–protein interactions. Some minor effects, such as short-range hydrogen bond interactions and electric dipole moments, can also play subtle roles in regulating the binding kinetics, and the improvement of our method will depend on how these factors are incorporated into the CG model. Another factor potentially affecting the accuracy of our method is the criteria used to determine the formation of an encounter complex. In our present model, the same criteria were applied to all protein complexes. However, as indicated in the transient-complex model, each protein complex has a unique binding interface and energy landscape, meaning that the criteria for the formation of different encounter complexes should be individually determined^63^. Thus, the use of different binding criteria for the formation of specific protein complexes would be expected to result in the improvement of our method. Finally, it is worth mentioning that changes to the experimental environments, such as the pH value of the solvent, the ion strength and the concentration of proteins, can lead to different measured values of *k*_*on*_. The sensitivity of these factors to simulations needs to be evaluated on a systematic level.

Another issue rises from the use of the decision tree method as a “black box”. A decision tree is a series of Boolean tests that serve to classify the data. The structure of a decision tree consists of a root node, a set of internal nodes, branches and leaves. The classification algorithm starts from the construction of the tree, in which one of the input indicators is selected as the root node and the training set is divided into two or more subsets. Additional partitions are carried out by generating new internal nodes. The branches coming out of the root and internal nodes are labeled with possible values of the indicators, while the leaves correspond to a decision, in this case, whether the *k*_*on*_ of a protein complex is overestimated or not. The most commonly used learning rules that are used to partition decision trees are based on the maximum entropy or largest information gain. After the tree construction, new data points can be traversed through the tree from the root to one of the leaf nodes, from which the class of each data point can be determined. We used the software *MATLAB* to automatically construct and optimize the tree after providing types of input indicators and training datasets. Consequently, the details of the learning procedure were not revealed, and the criteria of classification cannot be fully understood. The future application of machine learning programs that require more manual involvement is therefore needed to understand the mechanistic details of the overestimation.

Nevertheless, the use of reduced representations enables us to tackle the problems of protein–protein interactions with spatiotemporal ranges that are beyond the accessibility of the all-atom model. Therefore, our CG model can be applied to biological systems that are difficult to study using previous methods. For example, we will be able to study the binding kinetics of proteins with domains separated by flexible loops^78^. The interaction between thrombin and its functional inhibitor, rhodniin, was used as a test system in our previous study. We captured the conformational changes of the inter-domain loops by mapping the changes with time in the CG internal coordinates from the all-atom molecular dynamic simulations. We found that the association with full-length flexible rhodniin was faster than that with its two individual domains. This supports the idea of the existence of a “fly-casting” mechanism in which the partial structures of an intrinsic disordered protein first dock to the target, and then the remaining segments undergo conformational searches and sequentially coalesce around the target.

We can also extend our model to study the interaction between membrane proteins. Compared with the soluble proteins, it is technically much more difficult to simulate the association of membrane proteins due to the complexity of membrane environments. Furthermore, the binding of membrane proteins (2D) is measured in units that are different from those for the binding of soluble proteins (3D)^79^. The units of 2D *k*_*on*_ and *K*_*d*_ are reflected by the surface density of the interacting molecules and are expressed in terms of μm^2^/s and molecules/μm^2^, while the units of the 3D *k*_*on*_ and *K*_*d*_ are reflected by volumetric concentrations and are expressed by M^−1^ s^−1^ and M, if the first-order reaction is considered in which one ligand binds to only one receptor. This difference in units makes it very difficult to directly compare the 2D binding with the 3D. In one of our previous studies, we applied a similar method of coarse-grained kinetic Monte-Carlo simulation to study the binding of membrane receptors on cell surfaces. Using the interaction between the membrane proteins CD2 and CD58, two cell adhesion molecules known to mediate the activation of T cells and natural killer cells, as a test system, we showed that the 3D and 2D association rates could be directly linked and quantitatively compared^80^. However, a number of important factors were not considered in this simplified model. For instance, the fluctuations of the plasma membrane were only modeled implicitly. Moreover, in studies of both thrombin/rhodniin and CD2/CD58, the Go-like potential^81^,^82^,^83^ was used to characterize the binding between two interacting proteins; this potential is biased towards the formation of the native structure and will be replaced by the physics-based force field used in this paper to provide more accurate tests with greater predictive power.

Finally, we emphasized the importance of the protein conformational flexibility in regulating the protein association by assuming that the lack of conformational flexibility in the KMC is one of the reasons that led to the overestimation of calculated *k*_*on*_ values. It is worth mentioning that there are other possibilities that might also cause the overestimation. For instance, the calculation of the rate constant might be affected by omitting the degrees of freedom in the coarse-grained representation of the protein, as described in a previous study^84^. Moreover, in our KMC approach, the simulation will be terminated upon the formation of a ligand–receptor encounter complex, as we did not take into account the process of complex dissociation. Neglecting the effect of *k*_*off*_ might potentially lead to the systematic overestimation of *k*_*on*_. However, the simulation of protein-protein dissociation is an extremely challenging topic, especially when the protein complexes are energetically stable. It will take very large computational resources to obtain the process of dissociation for a protein complex with a long life span. Fortunately, a multi-scaling modeling framework can be used to estimate both *k*_*on*_ and *k*_*off*_ in simulation^85^. The framework can be constructed by combining the KMC simulation method with a previously developed rigid body-based simulation approach^86^ by feeding the kinetic information derived from the current CG model into rigid body-based lower-resolution simulations. Consequently, both long time-scale and high spatial-resolution, the information that is needed for the evaluation of the protein association and dissociation, can be captured in the model. This integrated procedure should help us to further understand the mechanisms of subcellular processes, such as complex assembly and membrane receptor clustering.

## Model and Methods

### Training and testing datasets of protein complexes used in the study

In this study, two sets of protein complexes were used to test the KMC simulations and to train and test the machine learning-based classification model. First, we used a set of 49 protein complex structures collected by Qin *et al*.^64^, consisting of 2 or more protein chains and including enzyme/inhibitor, ligand/receptor, regulator/effector, and other classes of protein interactions; the data included the PDB ids, experimentally observed association rate constants, and ionic strength used in the experiments. The experimentally measured rate constants ranged from 2.5 × 10^4^ to 1.3 × 10^9^ M^−1^ s^−1^. Detailed information for this benchmark set is listed in Supporting Information Tables S2A and S3A.

To avoid over-fitting during the machine learning of the 49 protein complex structures in the benchmark set, we collected another independent test set of 10 protein complexes from the SKEMPI database^87^. Complexes that had been included in the data set of the original 49 complexes and their homologs were excluded. In addition, only wild-type complexes were selected. Detailed information about the PDB ids of these complexes, experimentally observed association rate constants, and the ionic strength used in the experiments is listed in Tables S2B and S3B.

### Representation of the model

**Figure 7:**
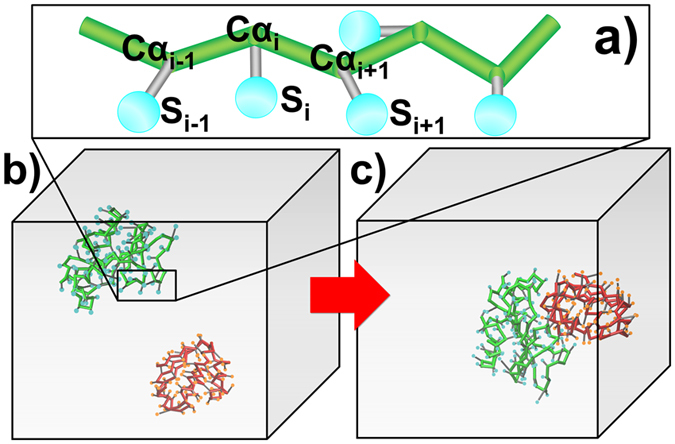
(**a**) Representation of our coarse-grained model. Each residue is represented by two sites, C and S. The positions of the Cα atoms (C) show the pseudo-backbone of the protein (green). The side chain of each residue is simplified as a representative center (S) (cyan) selected based on the specific properties of a particular amino acid. (**b**,**c**) A KMC simulation trajectory is initiated starting from a conformation in which a pair of proteins is randomly placed in a 3D cubic box (**b**), and the simulation is terminated if an encounter complex is formed between these two molecules (**c**).

The atomic structure of proteins was reduced to the following simplified model in the present simulations. Each residue is represented by two sites (Fig. 7a): one is the position of its C_α_ atom, while the other, indicated as S, is the representative center of a side-chain selected based on the specific properties of a given amino acid (Table S4). Similar representation has been used before to describe the structure and energetics of proteins^88^. Specifically, the representative centers of the side chains for charged residues were represented by their tip atoms to increase the sensitivity of the electrostatic effect. The position of atom NZ was selected as the representative center for lysine, while the centers of atoms NH1 and NH2 were selected as the representative centers for arginine, the centers of atoms OD1 and OD2 as the representative centers for aspartic acid, the centers of atoms OE1 and OE2 as the representative centers for glutamic acid, and the centers of atoms CG, ND1, CD2, CE1, and NE2 as the representative centers for histidine. The representative centers of all other amino acids are described either by the outmost atom on the side chains, such as serine and threonine, or the centers of a group of selected atoms which are located at the outer end of the side chains, such as the amino acids with aromatic rings. The detailed description of representative centers for all amino acids can be found in Table S4.

In addition, because each residue only contains two sites in this CG representation, the computational mutation of a specific residue in the barnase/barstar complex was accomplished as follows. For each of the 11 mutants in the test set, one or two charged residues were replaced by alanine. Computationally, the coordinates of all the side chain atoms of the original charged residues except their C_β_ atoms were truncated. The C_β_ atoms became the new side-chain function centers of the mutated alanine, and the charge of the side chains was neutralized.

### The total energy of interaction between the two proteins

The total energy of interaction between two proteins during association (*E*_*tot*_) described by a simple physics-based potential function consisting of three terms that can be written as


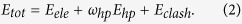


The first component on the right side of equation (2) is the electrostatic interaction, previously used in the Kim-Hummer model^89^,^90^:


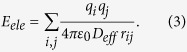


The Kim-Hummer potential, together with an intramolecular Go-like potential, was developed to model flexible protein interactions^91^. In equation (3), q_i_ is the charge of residue i. At pH 7, q_i_ equals +e for Lys and Arg, −e for Asp and Glu, and +0.5e for His (e is the elementary charge). The charge was assigned to the representative center of the side-chain of each corresponding residue. ε_0_ is the vacuum electric permittivity. An effective dielectric coefficient, *D*_*eff*_ = *D*_*s*_ exp(*r*_*ij*_/*ξ*), is used to reflect the shielding effect between two residues in which the representative centers of the side-chain are separated by a distance of r_ij_. As described in a previous study^90^, *D*_*s*_ = 10 is used to describe the local dielectric environment in which two proteins form an interface, and ξ is the Coulomb Debye length used to mimic the screening effect at different ion strengths, as discussed in the Results. The profiles of the electrostatic potential at different ionic strengths are plotted in Fig. S1, compared to the box size. It is worth mentioning that the Coulomb potential between charged atoms has also been used to model the binding between protein and DNA molecules^92^,^93^.

The second component, E_hp_, is the hydrophobic interaction, which is calculated by summing the hydrophobic scores of all contact residue pairs (residue i in chain 1 and residue j in chain 2) in which the representative centers of the side-chain are close to each other (r_ij_ < 6 Å)^94^ and can be expressed as


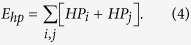


The hydrophobic scores of a contact residue pair, *HP*_*i*_ and *HP*_*j*_, were taken from a previous study by Kyte and Doolittle^95^. The value of the constant w_hp_, which is used to re-scale the weights of the energy terms and determine the relative contributions between the hydrophobic and electrostatic interactions, is 0.04.

Finally, the excluded volume effect during protein binding is taken into account in the third component of equation (2):


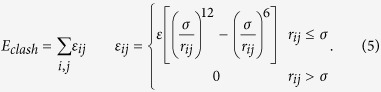


The depth of the potential ε equals 5 kT. The value of σ defines the finite distance at which the inter-particle potential is zero; it is set as 3.8 Å between two Cα atoms, 2.8 Å between a Cα atom and the representative center of a side-chain, and 2.2 Å between the representative centers of two side-chains.

### The kinetic Monte-Carlo (KMC) simulation algorithm

**Figure 8:**
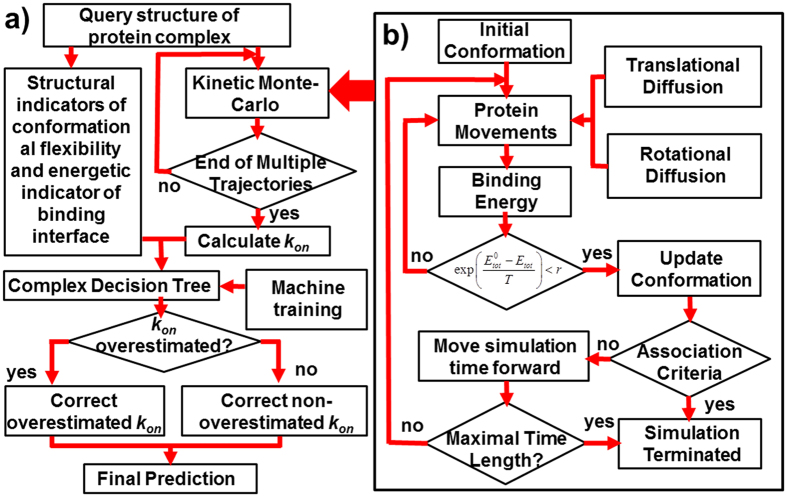
(**a**) Flowchart of the overall prediction framework, in which multiple trajectories of the KMC simulation are used to calculate *k*_*on*_. In parallel, three indicators are calculated based on the structural and energetic features at the binding interface of the query protein complex. These indicators are input into a trained “complex decision tree” to identify potential overestimation, and then the *k*_*on*_ calculated from the KMC simulations is adjusted based on the machine learning output. (**b**) Procedures involved in the KMC simulation. The detailed simulation algorithm is described in the Methods.

The association of two proteins was simulated using the KMC algorithm (Fig. 8b). The simulation was initiated starting from an orientation in which a pair of CG structural models of interacting proteins was randomly placed in a 3D cubic box (10 × 10 × 10 nm, i.e., the concentration is equal to 1.67 mM) (Fig. 7b). After the initial orientation was randomly generated, both proteins randomly diffused in the simulation box. The translational and rotational diffusion constants were obtained by fitting data calculated using a precise boundary element method^96^,^97^. The values of the diffusion constants for all test proteins are listed in Table S2A,B. The translational and rotational diffusion of the proteins was performed in a similar way to that in our previous study^86^. In detail, the internal degrees of freedom were fixed for both proteins, and each protein moved as a rigid body. In other words, the coordinates of a molecule only changed along the three translational and three rotational degrees of freedom, while the structural parameters of the molecule, such as the bond angles and bond dihedrals, remained unchanged. More detailed operations are described as follows. For translations, the probability of diffusion and the translation distance, in which each molecule moves in a random direction with a random length *r* (the average distance of diffusion is 10 Å), were computed in each simulation time step, Δt (1 ns). The probability of diffusion is


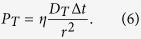


In equation (6), η equals 6 for diffusion in three dimensions, and *D*_*T*_ is the translational diffusion constant of the selected protein. A periodic boundary condition was applied to any protein that reached the boundary of the simulation box. The rotational movement was then calculated after the translational movement. For rotations, within each time step, the molecule randomly rotates around each Euler angle with a value of 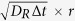, where *D*_*R*_ is the rotational diffusion constant of the molecule and r a randomly generated number between −1 and 1.

After the calculation of the translational and rotational movements for both proteins in the system, the energy between the two proteins was calculated using equation (2). The probability of acceptance of the diffusion *p* is calculated using the function^98^


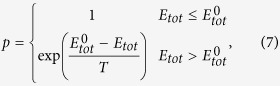


where 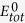 and *E*_*tot*_ are the total energy of the system before and after diffusion, respectively. The decision on whether diffusion occurred was made by comparing a generated random number with the calculated probability. At the end of each simulation step, the distances between all intermolecular interfacial pairs were calculated to determine how many native contacts were recovered. A native contact is defined as a pair of residues, i and j, in a native complex, the interaction of which contributes significantly to the total energy (*E*_*ij*_ < −1 kT) of the complex. A native contact was considered to be recovered during simulation if the distance between the representative centers of the two residues was less than 2 Å from the distance in the native conformation. The numbers of native contacts for each complex in the 2 benchmark sets are listed in Table S2A,B. When at least three native contacts were recovered, we assumed that the two proteins formed an encounter complex and the current simulation trajectory was terminated (Fig. 6c). Otherwise, the simulation ended when it reached the predefined maximal duration of 1000 ns.

### Calculation of the protein association rate constant from the KMC simulations

Multiple trajectories of KMC simulations were generated for each protein complex. The rate constant of the protein association was derived by counting how many protein complexes were associated from these simulation trajectories. The calculation of the association rate was based on the assumption that the formation of an encounter complex is rate-limiting (i.e., the transition from an intermediate encounter structure to its final native complex is much faster than the dissociation from the encounter complex). Each KMC simulation trajectory was terminated either when an encounter complex was formed or at the end of the simulation. After all N_tot_ simulation trajectories were completed, a success rate of ρ (ρ = N_on_/N_tot_) was derived, in which N_on_ is the number of times that two proteins form an encounter complex. Given the volume of the simulation box, V, the *k*_*on*_ of protein association can be written as


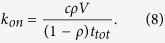


In equation (8), *t*_*tot*_ is the maximal simulation time for each trajectory and *c* is a constant that converts units from molecule/nm^3^ to M. The detailed derivation of equation (8) can be found in the Supporting Information.

### Identification of potentially overestimated rates using a machine learning algorithm

We observed that, in some cases, our *k*_*on*_ calculated from the KMC simulations was seriously overestimated compared to the experimental value. To identify potential overestimation, a machine learning algorithm was implemented to correct the simulation results (Fig. 8a). Before the algorithm was applied to a specific test, a training dataset was selected and classified into the predefined groups of overestimated and non-overestimated using the “complex decision tree”. The method is included as one of the “classification Learner” packages in *MATLAB*. Three indicators were chosen as inputs for each pair of proteins in the complex in the training set. The first two indicators take account of the conformational flexibility (the percentage of interface residues on flexible loops) of each of the two interacting proteins. The flexible loops are defined by the regions in the proteins whose secondary structural types are neither α-helix nor β-strand. The secondary structure type of a residue is determined by the standard DSSP algorithm by calculating the geometry of the hydrogen bonds in the backbone of a protein^99^. The third indicator is the energetic factor *r*_*elec*_, which is the ratio of the electrostatic potential at the binding interface to that of the whole protein pair. The ratio is defined as follows.


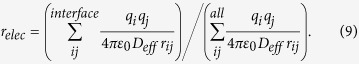


The numerator in the above equation is the summation over all residue pairs at the binding interfaces of a native protein complex. Residue i in chain 1 and residue j in chain 2 are at binding interfaces if the representative centers of the side-chains of these two residues are close to each other (r_ij_ < 6 Å) in the native structure of a protein complex. By contrast, the denominator in equation (9) is the summation over all residue pairs in a native protein complex. The definitions of all the other variables in equation (9) are the same as in equation (3). Two classes were designed as outputs: “overestimated” and “non-overestimated”. After the KMC simulations, if the calculated *k*_*on*_ of a protein complex in the training set was more than 4 times greater than the experimentally derived rate, it was assigned as “overestimated”. The same criterion of overestimation was used in a previous study^77^.

Based on the classification of all protein complexes in the training set, cutoff boundaries for the three input indicators were determined by machine learning. After the training, the calculated *k*_*on*_ for a new protein complex for which no experimental value is available was predicted as ‘overestimated” or “non-overestimated” based on the values of the three indicators for this protein complex (Fig. 8a). If the calculated *k*_*on*_ was predicted as “overestimated”, it was adjusted by dividing the original value by a correction factor, which is the geometric average of the calculated *k*_*on*_/experimental *k*_*on*_ ratio for all predicted overestimated protein complexes in the training set. Likewise, if the calculated *k*_*on*_ was predicted as “non-overestimated”, the geometric average of the calculated *k*_*on*_/experimental *k*_*on*_ ratio for all predicted non-overestimated protein complexes in the training set was used as the correction factor.

Two specific strategies were used to test the effect of this machine learning-based correction. They were applied to minimize the possibility that the model improvement was due to the result of data fitting through parameter adjustment. The first was the cross-validation of the 49-benchmark set. In this test, the strictest method, leave-one-out, was applied to avoid the potential over-fitting of the model. During each run of the leave-one-out test, one of the 49 protein complexes was selected as the test, while the remaining 48 were classified into “overestimated’ and “non-overestimated” groups and used as the training set, and the *k*_*on*_ for the test protein complex was adjusted by the training results. In the second strategy, a new independent 10-complex test set was constructed to further exclude bias in model training, and the 49-benchmark set was used as the training set to adjust the simulated *k*_*on*_ values for all protein complexes in the new test set. This second strategy further rules out the possibility of model over-fitting. The prediction results for the 49-complex training set (leave-one-out) and the results for the 10-complex testing set using the trained model are described in the Results.

### Calibration of computational performance

Because the cost of performing machine learning calculations is negligible after training, we only benchmark the computational performance of coarse-graining kinetic Monte-Carlo simulations. Specifically, two systems are used. The first is the protein complex barnase/barstar (1BRS), which we used as a test system to evaluate the robustness of our KMC simulation in our study. The second is B. anthracis Protective Antigen complexed with human Anthrax toxin receptor (1T6B), which is the largest system in the 49-complex benchmark set. As a result, for the system of 1BRS, it takes 12 seconds on average to generate a trajectory of 100 ns on a regular Linux desktop. For the system of 1T6B, it takes 130 seconds on average to generate a trajectory with the same length. Based on previous studies in the literature, it is shown that a typical 100 ns trajectory of BD simulation for a protein complex of normal size takes approximately an hour on a regular Linux desktop^100^,^101^. This indicates that our CG simulations are much faster than the traditional all-atom Brownian dynamic simulations.

### Availability of the simulation source codes

The source codes of this method for the protein–protein association rate constant prediction are available for download at: https://sourceforge.net/projects/pp-association-rate-prediction/. This package contains a set of Perl scripts for the batch prediction of the protein–protein association rates, a couple of executable files and their source codes, and a MATLAB prediction model to determine whether the predicted association rate for the target protein complex would be overestimated or not. It also offers a demonstration example of how to obtain the predicted association rate. These scripts work on a Linux platform, and downloading is free for academic users.

## Additional Information

**How to cite this article**: Xie, Z.-R. *et al*. Predicting Protein–protein Association Rates using Coarse-grained Simulation and Machine Learning. *Sci. Rep.* **7**, 46622; doi: 10.1038/srep46622 (2017).

**Publisher's note:** Springer Nature remains neutral with regard to jurisdictional claims in published maps and institutional affiliations.

## Supplementary Material

Supporting Information
