# Supplementary material for: Predicting Protein–protein Association Rates using Coarse-grained Simulation and Machine Learning
Source: Sci Rep. 2017 Apr 18;7:46622. doi: 10.1038/srep46622 (PMC5394550; doi:10.1038/srep46622)
Supplement: Supporting Information [file srep46622-s1.pdf]

# Predicting Protein-protein Association Rates using Coarse-grained Simulation and Machine Learning

Zhong-Ru Xie, Jiawen Chen, Yinghao Wu

# Supporting Information

**Table S1:** The Debye length at different ionic strengths

| $I$ [a] (mM) | ξ (Å) |
|---|---|
| 13 | 26.7 |
| 23 | 20.0 |
| 33 | 16.7 |
| 53 | 13.2 |
| 103 | 9.5 |
| 203 | 6.7 |
| 503 | 4.3 |

[a]I stands for ionic strength and ξ the Debye length; the data were collected by Alsallaq and Zhou (Alsallaq and Zhou, 2007)

**Table S2A:** The diffusion constants ($D_x$ and $D_r$) and the calculated ξ values at different ionic strengths for the 49 protein complexes

| PDB ID | M.W.1 (kDa) | M.W.2 (kDa) | $Dx_1$ [a] (Å$^2$/ns) | $Dx_2$ [a] (Å$^2$/ns) | $Dr_1$ [b] (Degree /ns) | $Dr_2$ [b] (Degree /ns) | $I$ [c] (M) | ξ (Å) | Native connections |
|---|---|---|---|---|---|---|---|---|---|
| 1eqy | 13.8 | 39.5 | 11.1 | 7.8 | 1.26 | 0.44 | 0.11 | 9.2 | 7 |
| 2j0t | 17.7 | 13.6 | 10.2 | 11.2 | 0.98 | 1.27 | 0.23 | 6.3 | 5 |
| 1jwh | 37.0 | 44.4 | 8.0 | 7.5 | 0.47 | 0.39 | 0.16 | 7.6 | 12 |
| 2ajf | 65.7 | 19.1 | 6.6 | 10.0 | 0.26 | 0.91 | 0.16 | 7.6 | 4 |
| 1kac | 20.4 | 13.6 | 9.8 | 11.2 | 0.85 | 1.27 | 0.16 | 7.6 | 10 |
| 2i25 | 12.5 | 14.2 | 11.5 | 11.0 | 1.38 | 1.22 | 0.16 | 7.6 | 7 |
| 1sbb | 26.2 | 26.3 | 9.0 | 9.0 | 0.66 | 0.66 | 0.16 | 7.6 | 9 |
| 1t6b | 74.4 | 18.7 | 6.3 | 10.0 | 0.23 | 0.93 | 0.05 | 13.6 | 20 |
| 2vir | 47.4 | 29.4 | 7.4 | 8.6 | 0.37 | 0.59 | 0.16 | 7.6 | 4 |
| 1kkl | 55.1 | 9.5 | 7.0 | 12.6 | 0.31 | 1.83 | 0.02 | 21.5 | 10 |
| 1mq8 | 20.2 | 19.5 | 9.8 | 9.9 | 0.86 | 0.89 | 0.16 | 7.6 | 7 |
| 1akj | 41.1 | 25.1 | 7.7 | 9.1 | 0.42 | 0.69 | 0.16 | 7.6 | 11 |
| 1gxd | 68.6 | 21.1 | 6.5 | 9.6 | 0.25 | 0.82 | 0.166 | 7.5 | 32 |
| 1uea | 18.3 | 19.9 | 10.1 | 9.8 | 0.95 | 0.87 | 0.23 | 6.3 | 17 |
| 1jtg | 28.8 | 18.1 | 8.7 | 10.1 | 0.6 | 0.95 | 0.025 | 19.2 | 20 |
| 1kxq | 54.6 | 13.2 | 7.0 | 11.3 | 0.32 | 1.31 | 0.206 | 6.7 | 11 |
| 1e6j | 47.2 | 23.1 | 7.4 | 9.4 | 0.37 | 0.75 | 0.16 | 7.6 | 4 |
| 2b42 | 40.0 | 20.2 | 7.8 | 9.8 | 0.43 | 0.86 | 0.1 | 9.6 | 10 |
| 1qa9 | 11.2 | 10.4 | 11.9 | 12.2 | 1.54 | 1.66 | 0.166 | 7.5 | 30 |
| 1a22 | 19.8 | 21.1 | 9.8 | 9.6 | 0.88 | 0.82 | 0.05 | 13.6 | 18 |
| 1e4k | 47.5 | 18.9 | 7.4 | 10.0 | 0.36 | 0.92 | 0.16 | 7.6 | 13 |
| 2b4j | 30.8 | 9.0 | 8.5 | 12.8 | 0.56 | 1.92 | 0.181 | 7.1 | 16 |
| 2vdb | 63.1 | 6.0 | 6.7 | 14.6 | 0.27 | 2.86 | 0.16 | 7.6 | 26 |
| 1ktz | 9.0 | 11.7 | 12.8 | 11.8 | 1.92 | 1.49 | 0.16 | 7.6 | 12 |
| 1gl1 | 26.3 | 3.7 | 9.0 | 17.2 | 0.66 | 4.63 | 0.11 | 9.2 | 8 |
| 1n8o | 26.3 | 15.0 | 9.0 | 10.8 | 0.66 | 1.16 | 0.21 | 6.6 | 8 |
| 1jps | 46.9 | 22.0 | 7.4 | 9.5 | 0.37 | 0.79 | 0.16 | 7.6 | 17 |
| 3bp8 | 83.7 | 8.2 | 6.1 | 13.2 | 0.21 | 2.1 | 0.223 | 6.4 | 7 |
| 1sgn | 20.4 | 5.6 | 9.8 | 15.0 | 0.85 | 3.09 | 0.26 | 6 | 6 |
| 1vfb | 24.5 | 14.2 | 9.2 | 11.0 | 0.71 | 1.22 | 0.15 | 7.8 | 3 |
| 1agr | 38.5 | 14.1 | 7.9 | 11.0 | 0.45 | 1.23 | 0.11 | 9.2 | 21 |
| 1fle | 26.4 | 5.2 | 8.9 | 15.4 | 0.66 | 3.35 | 0.25 | 6.1 | 14 |
| 2btf | 41.0 | 15.3 | 7.7 | 10.7 | 0.42 | 1.13 | 0.025 | 19.2 | 15 |
| 1lfd | 9.6 | 18.4 | 12.6 | 10.1 | 1.81 | 0.94 | 0.03 | 17.6 | 14 |
| 1oc0 | 40.0 | 4.1 | 7.8 | 16.7 | 0.43 | 4.26 | 0.165 | 7.5 | 6 |
| 1mah | 58.6 | 6.7 | 6.9 | 14.1 | 0.3 | 2.58 | 0.1 | 9.6 | 15 |
| 1ewy | 33.3 | 10.8 | 8.3 | 12.1 | 0.52 | 1.61 | 0.31 | 5.5 | 18 |
| 1bml | 27.5 | 35.0 | 8.8 | 8.1 | 0.63 | 0.5 | 0.1 | 9.6 | 36 |

| | | | | | | | | | |
|---|---|---|---|---|---|---|---|---|---|
| 1ffw | 14.1 | 7.5 | 11.0 | 13.6 | 1.23 | 2.32 | 0.15 | 7.8 | 18 |
| 4htc | 32.0 | 6.7 | 8.4 | 14.1 | 0.54 | 2.58 | 0.175 | 7.3 | 21 |
| 1emv | 9.1 | 14.4 | 12.7 | 11.0 | 1.9 | 1.2 | 0.25 | 6.1 | 12 |
| 1i2m | 18.1 | 42.7 | 10.1 | 7.6 | 0.95 | 0.41 | 0.045 | 14.3 | 31 |
| 1brs | 11.9 | 9.6 | 11.7 | 12.6 | 1.46 | 1.81 | 0.103 | 9.5 | 12 |
| 1udi | 25.0 | 9.1 | 9.1 | 12.7 | 0.69 | 1.9 | 0.08 | 10.7 | 22 |
| 2pcf | 10.9 | 27.5 | 12.0 | 8.8 | 1.59 | 0.63 | 0.1 | 9.6 | 18 |
| 1dfj | 13.6 | 50.2 | 11.2 | 7.2 | 1.27 | 0.35 | 0.2 | 6.8 | 19 |
| 1e6e | 50.3 | 12.4 | 7.2 | 11.5 | 0.34 | 1.39 | 0.21 | 6.6 | 27 |
| 7cei | 9.6 | 14.0 | 12.6 | 11.1 | 1.81 | 1.24 | 0.25 | 6.1 | 19 |
| 2pcc | 32.3 | 11.9 | 8.4 | 11.7 | 0.54 | 1.46 | 0.15 | 7.8 | 15 |

[a]$D_x$ was calculated using the equation: $D_x = 26.647/\sqrt[3]{M.W.}$ obtained by curve fitting to the data from reference (Aragon and Hahn, 2006).
[b]$D_r$ was calculated using the equation: $D_r = 17.33/M.W.$ obtained by curve fitting to the data from reference (Aragon and Hahn, 2006).
[c]I stands for ionic strength; the experimental data are from reference (Qin, et al., 2011)

**Table S2B:** The diffusion constants ($D_x$ and $D_r$) and the calculated ξ values at different ionic strengths for an independent test set of 10 protein complexes

| PDB ID | M.W.1 (kDa) | M.W.2 (kDa) | $Dx_1$ [a] (Å$^2$/ns) | $Dx_2$ [a] (Å$^2$/ns) | $Dr_1$ [b] (Degree /ns) | $Dr_2$ [b] (Degree /ns) | $I$ [c] (M) | ξ (Å) | Native connections |
|---|---|---|---|---|---|---|---|---|---|
| 1tlu | 12.9 | 12.9 | 11.4 | 11.4 | 1.35 | 1.35 | 0.01 | 30.4 | 30 |
| 1iar | 14.2 | 20.7 | 11.0 | 9.7 | 1.22 | 0.84 | 0.15 | 7.8 | 14 |
| 2b42 | 40.0 | 20.2 | 7.8 | 9.8 | 0.43 | 0.86 | 0.1 | 9.6 | 10 |
| 2wpt | 9.0 | 12.4 | 12.8 | 11.5 | 1.92 | 1.39 | 0.2 | 6.8 | 9 |
| 2vlq | 9.2 | 14.7 | 12.7 | 10.9 | 1.88 | 1.18 | 0.2 | 6.8 | 8 |
| 1rew | 22.7 | 9.5 | 9.4 | 12.6 | 0.76 | 1.83 | 0.5 | 4.3 | 13 |
| 1a4y | 50.6 | 13.5 | 7.2 | 11.2 | 0.34 | 1.28 | 0.1 | 9.6 | 32 |
| 1a22 | 19.8 | 21.1 | 9.8 | 9.6 | 0.88 | 0.82 | 0.025 | 19.2 | 18 |
| 1dan | 42.5 | 21.0 | 7.6 | 9.7 | 0.41 | 0.82 | 0.15 | 7.8 | 36 |
| 3hfm | 47.2 | 14.2 | 7.4 | 11.0 | 0.37 | 1.22 | 0.15 | 7.8 | 5 |

[a]$D_x$ was calculated using the equation: $D_x = 26.647/\sqrt[3]{M.W.}$ obtained by curve fitting to the data from reference (Aragon and Hahn, 2006).

[b]$D_r$ was calculated using the equation: $D_r = 17.33/M.W.$ obtained by curve fitting to the data from reference (Aragon and Hahn, 2006).

[c]Based on the experimental data in the SKEMPI database (Moal and Fernandez-Recio, 2012)

**Table S3A:** The 3 properties input to train the machine learning model and to predict whether the preliminary $k_{on}$ results produced by our kinetic Monte-Carlo simulation model were overestimated or not and the final $k_{on}$ results for the 49 complex training set

| PDB ID | $r_{elec}$[a] | %L1[b] | %L2[b] | Over-estimated | $k_{on}$ (exp.) ($M^{-1}s^{-1}$)[c] | $k_{on}$ (preliminary) ($M^{-1}s^{-1}$)[d] | *st.dev.*[g] | $k_{on}$ (final prediction) ($M^{-1}s^{-1}$)[e] |
|---|---|---|---|---|---|---|---|---|
| 1eqy | -0.11 | 29.4 | 67.7 | n | 2.50E+04 | 3.59E+04 | 3.10E+04 | 9.30E+04 |
| 2j0t | 0.73 | 74.2 | 57.1 | n | 5.20E+04 | 2.40E+04 | 3.10E+04 | 3.66E+04 |
| 1jwh | 0.70 | 71.4 | 66.7 | o | 6.70E+04 | 7.79E+05 | 5.70E+05 | 1.65E+06 |
| 2ajf | 1.28 | 33.3 | 75.0 | n | 7.10E+04 | 5.99E+03 | 1.90E+04 | 1.55E+04 |
| 1kac | 1.56 | 86.4 | 70.4 | o | 7.30E+04 | 3.13E+06 | 1.33E+06 | 2.78E+05 |
| 2i25 | 2.59 | 66.7 | 66.7 | o | 9.00E+04 | 2.10E+06 | 1.25E+06 | 1.87E+05 |
| 1sbb | 4.51 | 66.7 | 77.8 | o | 1.00E+05 | 3.74E+06 | 1.54E+06 | 3.32E+05 |
| 1t6b | 3.25 | 84.2 | 63.3 | o | 1.10E+05 | 8.63E+06 | 3.03E+06 | 7.67E+05 |
| 2vir | 0.98 | 73.7 | 46.2 | n | 1.10E+05 | 3.59E+05 | 5.06E+05 | 5.50E+05 |
| 1kkl | -5.17 | 19.4 | 64.5 | o | 1.30E+05 | 5.39E+05 | 6.61E+05 | 4.80E+04 |
| 1mq8 | -2.24 | 47.8 | 100.0 | o | 1.30E+05 | 8.39E+05 | 9.49E+05 | 7.46E+04 |
| 1akj | 1.53 | 62.9 | 69.8 | o | 1.40E+05 | 5.56E+06 | 1.37E+06 | 8.50E+06 |
| 1gxd | 1.42 | 73.8 | 77.8 | o | 1.40E+05 | 5.38E+06 | 1.56E+06 | 4.78E+05 |
| 1uea | 0.95 | 81.4 | 77.8 | o | 2.00E+05 | 1.02E+06 | 4.05E+05 | 9.07E+04 |
| 1jtg | 3.10 | 46.5 | 52.3 | o | 2.20E+05 | 5.62E+06 | 1.63E+06 | 5.00E+05 |
| 1kxq | 5.01 | 90.0 | 65.7 | o | 2.40E+05 | 2.22E+06 | 8.07E+05 | 1.98E+05 |
| 1e6j | 0.48 | 66.7 | 29.4 | n | 3.50E+05 | 1.32E+05 | 8.88E+04 | 3.42E+05 |
| 2b42 | 3.82 | 53.1 | 35.7 | o | 3.60E+05 | 1.44E+06 | 8.13E+05 | 1.28E+05 |
| 1qa9 | 2.16 | 50.0 | 68.2 | o | 4.00E+05 | 6.44E+07 | 6.86E+06 | 5.73E+06 |
| 1a22 | 3.16 | 21.7 | 69.1 | o | 4.00E+05 | 1.86E+06 | 1.04E+06 | 1.66E+05 |
| 1e4k | 1.00 | 84.9 | 79.2 | o | 4.20E+05 | 8.56E+06 | 2.30E+06 | 7.62E+05 |
| 2b4j | 2.12 | 14.3 | 66.7 | o | 4.80E+05 | 3.01E+07 | 3.18E+06 | 2.67E+06 |
| 2vdb | 4.71 | 14.8 | 28.0 | o | 5.50E+05 | 3.65E+07 | 5.91E+06 | 3.24E+06 |
| 1ktz | 1.33 | 84.6 | 50.0 | o | 7.40E+05 | 5.00E+07 | 4.15E+06 | 4.44E+06 |
| 1gl1 | -1.56 | 62.5 | 30.4 | o | 8.00E+05 | 1.64E+07 | 4.99E+06 | 3.86E+07 |
| 1n8o | 0.00 | 62.5 | 72.7 | n | 8.90E+05 | 2.40E+05 | 4.02E+04 | 2.46E+04 |
| 1jps | 2.03 | 65.6 | 47.2 | o | 9.80E+05 | 1.17E+07 | 2.83E+06 | 1.04E+06 |
| 3bp8 | -1.16 | 60.7 | 68.2 | - | 1.00E+06 | No Output[f] | | No Output[f] |
| 1sgn | -7.57 | 53.3 | 68.8 | n | 1.20E+06 | 1.32E+05 | 6.83E+04 | 1.17E+04 |
| 1vfb | -1.60 | 91.7 | 66.7 | - | 1.40E+06 | No Output[f] | | No Output[f] |
| 1agr | 2.81 | 70.4 | 51.7 | o | 1.70E+06 | 1.69E+07 | 3.18E+06 | 1.50E+06 |
| 1fle | 1.21 | 67.7 | 66.7 | n | 3.60E+06 | 9.60E+05 | 7.60E+05 | 8.53E+04 |
| 2btf | 1.73 | 54.6 | 31.4 | n | 6.60E+06 | 1.15E+07 | 2.68E+06 | 2.98E+07 |
| 1lfd | 1.64 | 31.3 | 36.8 | n | 7.70E+06 | 1.76E+07 | 4.11E+06 | 4.54E+07 |
| 1oc0 | 0.89 | 34.8 | 81.3 | n | 1.40E+07 | 8.39E+05 | 8.59E+05 | 2.17E+06 |
| 1mah | 0.83 | 72.1 | 65.2 | o | 1.50E+07 | 1.58E+08 | 1.09E+07 | 3.35E+08 |

| | | | | | | | | |
|---|---|---|---|---|---|---|---|---|
| 1ewy | 1.34 | 57.7 | 72.0 | n | 4.00E+07 | 9.85E+07 | 9.93E+06 | 2.55E+08 |
| 1bml | 1.37 | 78.3 | 56.4 | n | 5.40E+07 | 7.03E+06 | 1.98E+06 | 1.07E+07 |
| 1ffw | 1.49 | 29.4 | 52.6 | n | 6.20E+07 | 5.08E+07 | 5.54E+06 | 1.31E+08 |
| 4htc | 1.21 | 67.8 | 69.7 | n | 7.50E+07 | 7.33E+06 | 1.31E+06 | 6.52E+05 |
| 1emv | 0.93 | 53.9 | 66.7 | n | 9.00E+07 | 1.52E+07 | 3.13E+06 | 3.94E+07 |
| 1i2m | 1.27 | 56.4 | 84.6 | n | 1.00E+08 | 4.13E+07 | 4.22E+06 | 1.07E+08 |
| 1brs | 1.56 | 61.5 | 45.8 | n | 1.20E+08 | 4.42E+07 | 7.26E+06 | 1.14E+08 |
| 1udi | 0.46 | 75.0 | 25.8 | n | 1.50E+08 | 7.42E+07 | 7.88E+06 | 1.13E+08 |
| 2pcf | 0.93 | 75.8 | 63.3 | n | 1.80E+08 | 2.09E+08 | 1.75E+07 | 3.20E+08 |
| 1dfj | 1.37 | 57.8 | 52.9 | n | 3.40E+08 | 8.32E+06 | 2.80E+06 | 2.15E+07 |
| 1e6e | 1.15 | 24.3 | 72.7 | n | 4.00E+08 | 1.37E+08 | 1.10E+07 | 3.54E+08 |
| 7cei | 0.93 | 53.9 | 66.7 | n | 7.60E+08 | 1.18E+08 | 1.10E+07 | 3.07E+08 |
| 2pcc | 0.88 | 66.7 | 55.6 | n | 1.30E+09 | 1.25E+08 | 1.11E+07 | 3.23E+08 |

[a]$r_{elec}$ represents the ratio between the interfacial electrostatic energy and total electrostatic energy
[b]%L1 and %L2 represent the percentages of interfacial residues located on flexible loops in protein 1 and 2
[c]Experimental $k_{on}$ values collected by Qin et al. (Qin, et al., 2011)
[d]The $k_{on}$ s calculated using our kinetic Monte-Carlo model
[e]The final predicted $k_{on}$s after adjustment according to the cross-validation models
[f]There is no output of association between 2 subunits in any trajectory collected
[g]To calculate standard deviations, $10^4$ KMC simulation trajectories were generated for each protein complex. We randomly divided these trajectories into 10 groups. Each group contains $10^3$ trajectories. We estimated $k_{on}$ from $10^3$ trajectories of each group and derived 10 individual $k_{on}$s. The standard deviation was calculated from the group of $k_{on}$s.

**Table S3B:** The 3 properties input to train the machine learning model and to predict whether the preliminary $k_{on}$ results produced by our kinetic Monte-Carlo simulation model were overestimated or not and the final $k_{on}$ results for the 10 complex independent test set

| PDB ID | $r_{elec}$[a] | %L1[b] | %L2[b] | Over-estimated | $k_{on}$ (exp.) ($M^{-1}s^{-1}$)[c] | $k_{on}$ (preliminary) ($M^{-1}s^{-1}$)[d] | *st.dev.*[h] | $k_{on}$ (final prediction) ($M^{-1}s^{-1}$)[e] | $k_{on}$ (Trancompt) ($M^{-1}s^{-1}$)[f] |
|---|---|---|---|---|---|---|---|---|---|
| 1tlu | 4.12 | 15.2 | 20.8 | n | 5.60E+06 | 5.88E+06 | 2.71E+06 | 2.95E+05 | 4.70E+02 |
| 1iar | 1.05 | 4.3 | 69.6 | o | 1.30E+07 | 7.37E+07 | 7.46E+06 | 2.17E+08 | 2.23E+07 |
| 2b42 | 3.82 | 53.1 | 35.7 | n | 3.61E+05 | 7.20E+05 | 7.38E+05 | 3.62E+04 | No Output[g] |
| 2wpt | 1.07 | 50.0 | 64.3 | n | 5.00E+07 | 1.03E+08 | 9.75E+06 | 3.03E+08 | 4.90E+07 |
| 2vlq | 0.92 | 48.1 | 64.0 | n | 7.91E+07 | 1.34E+07 | 2.81E+06 | 3.93E+07 | 1.20E+08 |
| 1rew | 0.93 | 38.5 | 58.3 | n | 6.00E+05 | 3.60E+05 | 4.19E+05 | 1.06E+06 | 1.60E+06 |
| 1a4y | 1.07 | 60.4 | 53.5 | n | 2.80E+08 | 6.55E+07 | 7.59E+06 | 1.93E+08 | 1.16E+06 |
| 1a22 | 3.16 | 21.7 | 69.0 | o | 1.50E+05 | 1.20E+06 | 9.84E+05 | 6.04E+04 | 1.42E+05 |
| 1dan | 2.59 | 48.1 | 47.6 | o | 1.60E+05 | 1.86E+06 | 1.29E+06 | 9.37E+04 | No Output[g] |
| 3hfm | 1.15 | 92.9 | 74.1 | n | 1.49E+06 | 2.40E+05 | 3.10E+05 | 1.21E+04 | 7.89E+04 |

[a]$r_{elec}$ represents the ratio between the interfacial electrostatic energy and total electrostatic energy
[b]%L1 and %L2 represent the percentages of interfacial residues located on flexible loops in protein 1 and 2
[c]Based on experimental data in the SKEMPI database (Moal and Fernandez-Recio, 2012)
[d]The $k_{on}$ s calculated using our kinetic Monte-Carlo model
[e]The final predicted $k_{on}$s after adjustment according to the cross-validation models
[f]The predicted results from the TransComp server
[g]No prediction provided by TransComp server
[h]To calculate standard deviations, $10^4$ KMC simulation trajectories were generated for each protein complex. We randomly divided these trajectories into 10 groups. Each group contains $10^3$ trajectories. We estimated $k_{on}$ from $10^3$ trajectories of each group and derived 10 individual $k_{on}$s. The standard deviation was calculated from the group of $k_{on}$s.

**Table S4:** Coordinates, charge and hydrophobicity of side-chain function centers

| Amino acid | Functional site | Formal charge | Hydrophobicity [a] |
|---|---|---|---|
| Aspartic acid | the center of atom OD1 and OD2 | -1 | 0 |
| Glutamic acid | the center of atom OE1 and OE2 | -1 | 0 |
| Histidine | the center of atom CG, ND1, CD2, CE1 and NE2 | 0.5 | 0 |
| Lysine | atom NZ | 1 | 0 |
| Arginine | the center of atom NH1 and NH2 | 1 | 0 |
| Asparagine | the center of atom OD1 and ND2 | 0 | 0 |
| Glutamine | the center of atom OE1 and NE2 | 0 | 0 |
| Glycine | atom CA | 0 | 0 |
| Alanine | atom CB | 0 | 1.8 |
| Valine | the center of atom CG1 and CG2 | 0 | 4.2 |
| Leucine | the center of atom CD1 and CD2 | 0 | 3.8 |
| Isoleucine | atom CD1 | 0 | 4.5 |
| Threonine | the center of atom OG1 and CG2 | 0 | 0 |
| Methionine | atom CE | 0 | 1.9 |
| Cysteine | atom SG | 0 | 2.5 |
| Serine | atom OG | 0 | 0 |
| Tyrosine | atom OH | 0 | 0 |
| Phenylalanine | the center of atom CG, CD1, CD2, CE1, CE2 and CZ | 0 | 2.8 |
| Tryptophan | the center of atom CG, CD1, CD2, NE1, CE2, CE3, CZ2, CZ3 and CH2 | 0 | 0 |
| Proline | the center of atom CB, CG and CD | 0 | 0 |

[a]The hydrophobicity scores were assigned as in a previous study (Kyte and Doolittle, 1982). Negative hydrophobicity values were considered as 0 in the computation.

**The derivation of the rate constant calculation equation**

The computation of protein association rate constants is based on established theories and the consideration of an intermediate state (A*B) in which the two associating proteins A and B have a near-native separation and orientation (Schreiber, et al., 2009).

$$\mathrm{A} + \mathrm{B} \underset{\mathrm{k_b}}{\overset{\mathrm{k_f}}{\rightleftarrows}} \mathrm{A} * \mathrm{B} \overset{\mathrm{k_c}}{\rightarrow} \mathrm{AB} \qquad \text{(Eq. S1)}$$

The first step in protein association depends on the translation and diffusion of protein A and B towards each other. Based on the classical transition-state theory, the transition state is located at the top of the free-energy barrier separating A*B and C and the rate of generation of protein complex AB can be described as:

$$\frac{d[AB]}{dt} = k_{on}[A][B] = k_c[A * B] \qquad \text{(Eq. S2)}$$

In the equilibrium, the concentration the intermediate state (A*B) will be a constant,

$$\frac{d[A*B]}{dt} = k_f[A][B] - (k_b + k_c)[A * B] = 0 \qquad \text{(Eq. S3)}$$

From Eq. S3, the concentration of A*B can be described as:

$$[A * B] = \frac{k_f}{(k_b+k_c)}[A][B] \qquad \text{(Eq. S4)}$$

Combining Eq. S2 and Eq. S4,

$$\frac{d[AB]}{dt} = k_c[A * B] = \frac{k_c k_f}{(k_b+k_c)}[A][B] \qquad \text{(Eq. S5)}$$

If kc ≫ kb, then kon ≃ kf, and Eq. S5 approximates to

$$\frac{d[AB]}{dt} = k_{on}[A][B] \qquad \text{(Eq. S6)}$$

In our model, the initial concentration $[A_0]= [B_0]$, so,

$$[A_0] =[A]+[AB]=[B]+[AB] \qquad \text{(Eq. S7)}$$

$$[A]=[B]= [A_0 -AB] \qquad \text{(Eq. S8)}$$

Eq. S6 can be re-written as:

$$\frac{d[AB]}{dt} = k_{on}[A_0 - AB]^2 \qquad \text{(Eq. S9)}$$

Then

$$-\frac{d[A_0-AB]}{dt} = k_{on}[A_0 - AB]^2 \qquad \text{(Eq. S10)}$$

So

$$-\frac{d[A_0-AB]}{[A_0-AB]^2} = k_{on}dt \qquad \text{(Eq. S11)}$$

And

$$k_{on} = \frac{\frac{1}{[A_0]-[AB]} - \frac{1}{[A_0]}}{t_{tot}} = \frac{\frac{[AB]}{([A_0]-[AB])[A_0]}}{t_{tot}} \qquad \text{(Eq. S12)}$$

In our simulation, we generated multiple trajectories using our KMC model. After all $N_{tot}$ simulation trajectories were completed, a success rate $\rho$ ($\rho=N_{on}/N_{tot}$) was derived. $1/V = [A_0] = [A] + [AB] = [B] + [AB]$ (when the initial concentration $[A_0]= [B_0]$). $\rho = N_{on}/N_{tot} = [AB]/[A_0]$, and $(1-\rho) = ([A_0]- [AB])/[A_0]$. In addition, we need a constant $c$ to convert the units from molecule/nm$^3$ to M.

$$k_{on} = \frac{c\frac{[AB]}{([A_0]-[AB])[A_0]}\frac{1}{}}{t_{tot}} = \frac{c\frac{[AB]}{[A_0]}\frac{1}{[A_0]}}{\frac{([A_0]-[AB])}{[A_0]}t_{tot}} \qquad \text{(Eq. S13)}$$

Finally,

$$k_{on} = \frac{c\rho V}{(1-\rho)t_{tot}} \qquad \text{(Eq. S14)}$$

### Integrating the elastic network model (ENM) into KMC simulations

In the KMC simulation described in the paper, all the intramolecular degrees of freedom are fixed. Here we try to consider the effect of intramolecular flexibility on regulating binding kinetics by integrating the conformational variations into our simulation procedure. Our primary algorithm was slightly modified based on the coarse-grained nature of our model. Specifically, the ENM was used to modify the conformation of each pair of interacting proteins (Atilgan, et al., 2001; Bahar, et al., 2010). The ENM is based on the assumption that molecular vibrations near an equilibrium conformation can be determined by a coarse-grained harmonic potential:

$$V = \frac{\gamma}{2}\sum_{ij}\sigma_{ij}\left(\left|\vec{r}_{ij}\right| - \left|\vec{r}_{ij}^{\,0}\right|\right)^2, \sigma_{ij} = \begin{cases} 1 & \left|\vec{r}_{ij}^{\,0}\right| \le r_c \\ 0 & \left|\vec{r}_{ij}^{\,0}\right| > r_c \end{cases} \quad \text{(Eq. S15)}$$

Where $\left|\vec{r}_{ij}\right|$ and $\left|\vec{r}_{ij}^{\,0}\right|$ are the instantaneous and equilibrium values of the distance between Cα atoms *i* and *j*, γ is the uniform force constant, and the cutoff value, rc, is 13Å. We chose the ten lowest frequency modes for each protein. These modes describe the collective motions of the corresponding protein structure. The amplitude of these normal modes was arbitrarily determined within a comparable range of experimental temperature factors.

The integration of ENM into KMC simulations is relatively straightforward (dashed panels in **Fig S2**). Because the ENM only simulates small and fast conformational fluctuations near the equilibrium state of protein native structures, we can assume that the time scale of these motions is much faster than diffusion. Therefore, after translational and rotational diffusions are performed within each simulation time step, we randomly chose one of the lowest-frequency modes of the ENM for each pair of interacting proteins and changed their conformations along the selected mode.

In a proof-of-concept test, three different protein complexes were selected from the benchmark set to test the method. The overall testing results are plotted as a histogram in **Fig S3**. In this figure, the striped bars are calculated $k_{on}$s by the original KMC in which ENM was not integrated. The grey bars are calculated $k_{on}$s by the new KMC with ENM component described in the last paragraph, while the black bars are experimental $k_{on}$s. Using the original KMC, our calculated $k_{on}$s are smaller than the experimental values for protein complexes 1BRS and 1EMV, but much larger than the experimental values for protein complexes 1GXD. In another word, our prediction on 1GXD was overestimated. Using the KMC that contains conformational flexibility, we found that our newly calculated $k_{on}$s are closer to the experimental values. We found that the complex 1GXD has much higher degree of flexibility (75%) than the other two complexes at the binding interfaces, as shown in **Table S3A**. This explains the improvements in the new KMC. In the case of 1GXD, our simulations show that conformational fluctuation due to the high structural flexibility can impede association by creating additional entropy loss during binding.

Our preliminary testing results therefore support our hypothesis that the overestimation of was caused by the molecular flexibility of different proteins during association. A systematic analysis and benchmark test of the new simulation algorithm will be developed in the follow-up study. It is also worth of mentioning the limitation of the ENM approach, due to the fact that ENM takes a harmonic approximation to the energy surface of a protein which minimum is around its native conformation. Therefore, the ENM can only be used to model small conformational fluctuations.

**References**

Alsallaq, R. and Zhou, H.X. Prediction of protein-protein association rates from a transition-state theory. *Structure* 2007;15(2):215-224.

Aragon, S. and Hahn, D.K. Precise boundary element computation of protein transport properties: Diffusion tensors, specific volume, and hydration. *Biophys J* 2006;91(5):1591-1603.

Atilgan, A.R.*, et al.* Anisotropy of fluctuation dynamics of proteins with an elastic network model. *Biophysical Journal* 2001;80(1):505-515.

Bahar, I.*, et al.* Global dynamics of proteins: bridging between structure and function. *Annu Rev Biophys* 2010;39:23-42.

Kyte, J. and Doolittle, R.F. A simple method for displaying the hydropathic character of a protein. *Journal of molecular biology* 1982;157(1):105-132.

Moal, I.H. and Fernandez-Recio, J. SKEMPI: a Structural Kinetic and Energetic database of Mutant Protein Interactions and its use in empirical models. *Bioinformatics* 2012;28(20):2600-2607.

Qin, S., Pang, X. and Zhou, H.X. Automated prediction of protein association rate constants. *Structure* 2011;19(12):1744-1751.

Schreiber, G., Haran, G. and Zhou, H.X. Fundamental aspects of protein-protein association kinetics. *Chem Rev* 2009;109(3):839-860.

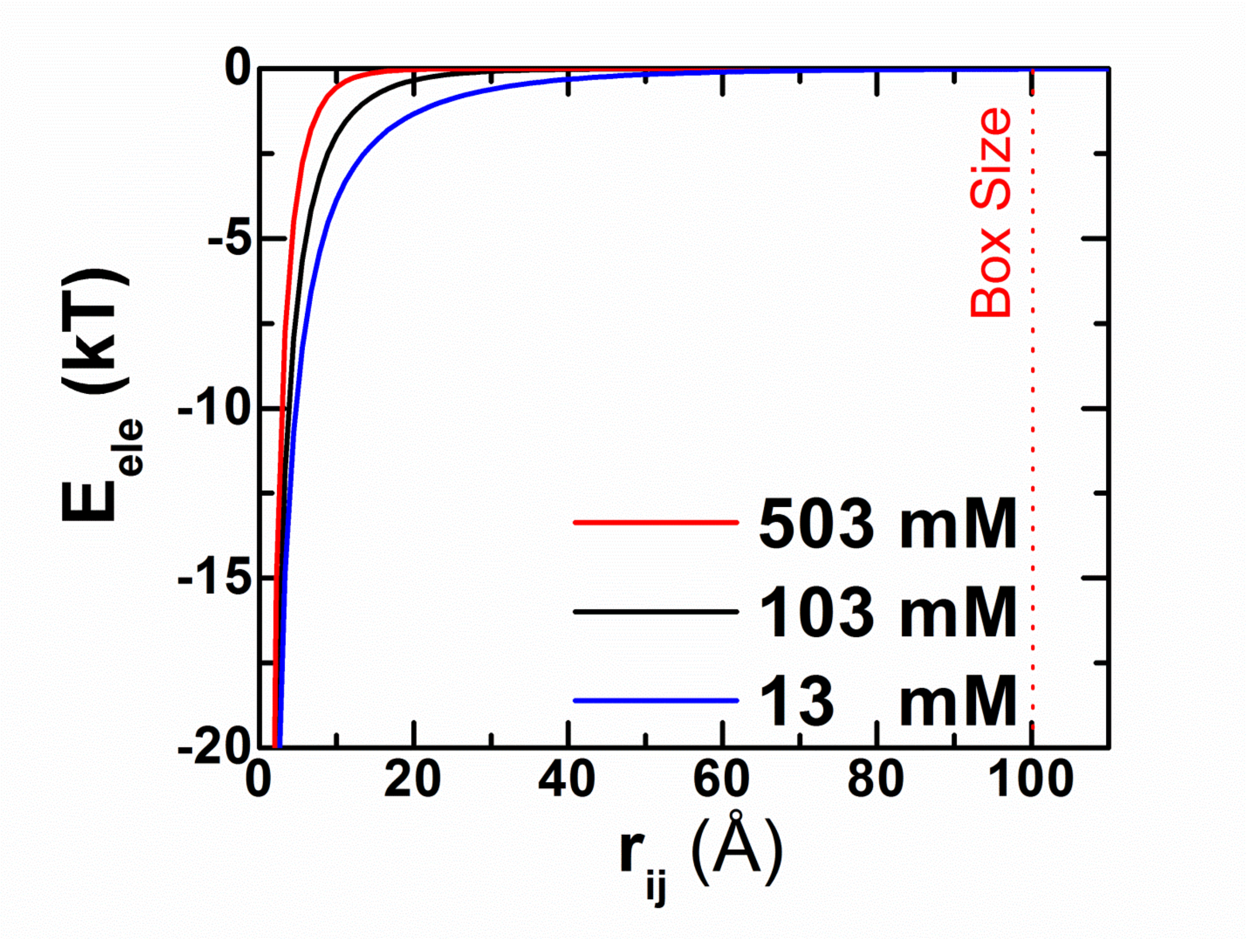


**Figure S1:** The profiles of the electrostatic potential are plotted at different ionic strengths. We used the Kim-Hummer model (equation (3)) to describe the electrostatic potential. The strength of potential is presented in the unit of kT. The red dashed line on the right side of the figure indicates the size of the simulation box (10nm). The figure shows that the electrostatic interactions have larger range under lower ionic strength than the higher strength, therefore accelerate the association of proteins.

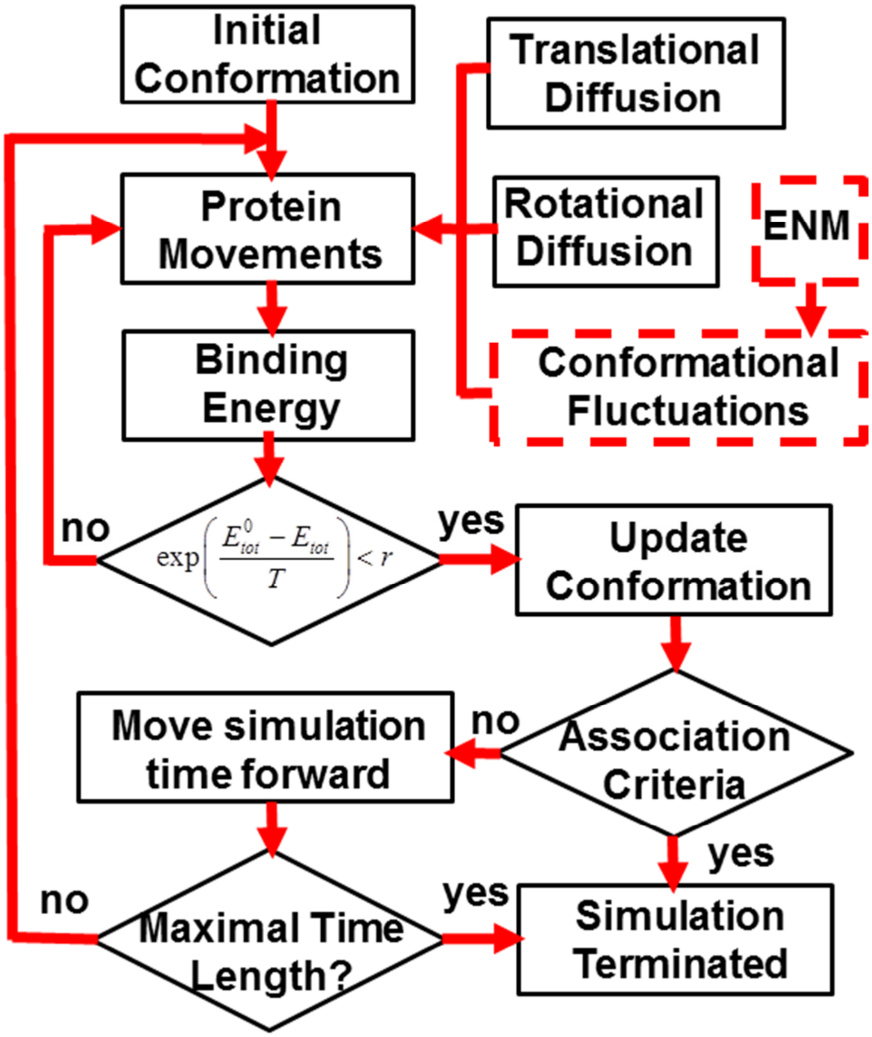


**Figure S2:** The flowchart of the KMC simulation by directly integrating the conformational fluctuations using elastic network model (ENM). The ENM component is highlighted in the dashed red panels.

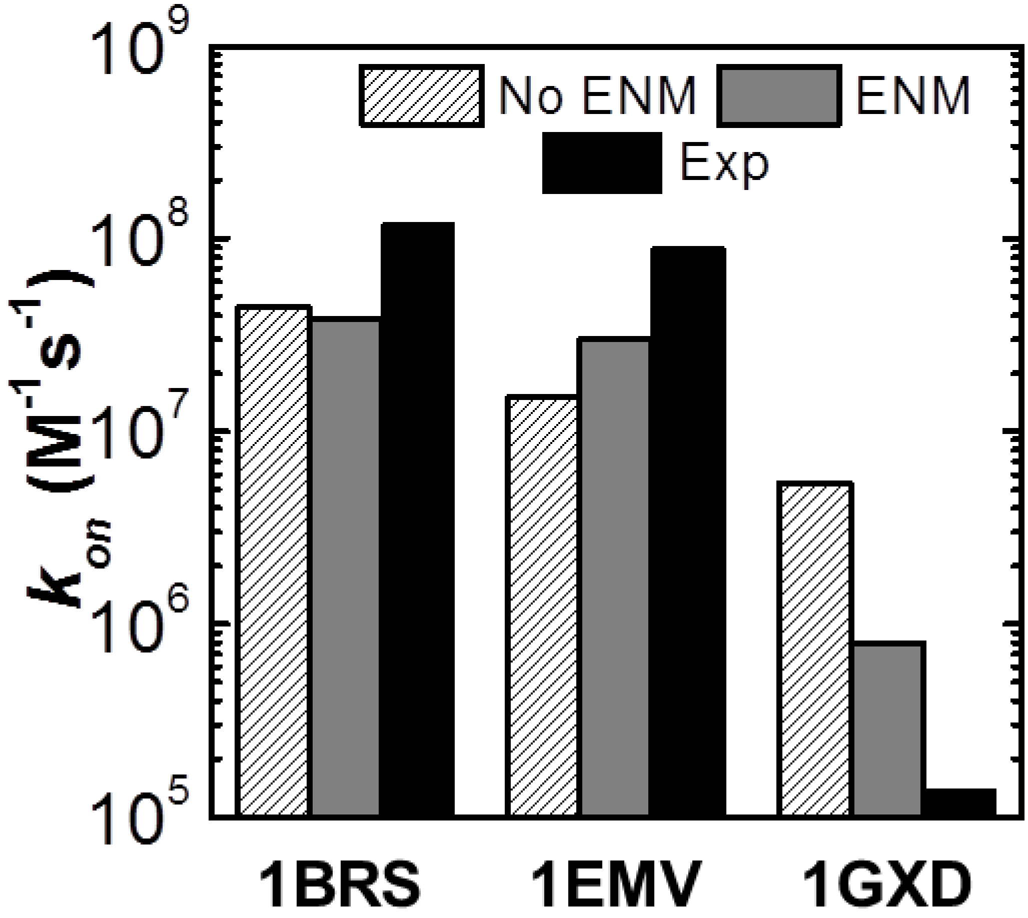


**Figure S3:** Three protein complexes were selected to test the new simulation with ENM, as indicated at the bottom of the figure. The striped bars are calculated $k_{on}$s by the original KMC in which ENM was not integrated. The grey bars are calculated $k_{on}$s by the new KMC with ENM component described in the last paragraph, while the black bars are experimental $k_{on}$s.
